# The Role of a Multidisciplinary Team in the Diagnosis and Treatment of Bone and Soft Tissue Sarcomas: A Single-Center Experience

**DOI:** 10.3390/jpm12122079

**Published:** 2022-12-16

**Authors:** Ke Pang, Xiaoning Guo, Tang Liu, Lu Wang, Ruiqi Chen, Zhiming Zhang, Lan Li, Yu He, Haixia Zhang, Songqing Fan, Chao Tu, Zhihong Li

**Affiliations:** 1 Department of Orthopedics, The Second Xiangya Hospital of Central South University, Changsha 410011, China; 2 Hunan Key Laboratory of Tumor Models and Individualized Medicine, The Second Xiangya Hospital of Central South University, Changsha 410011, China; 3 Department of Pathology, The Second Xiangya Hospital of Central South University, Changsha 410011, China; 4 Department of Radiology, The Second Xiangya Hospital of Central South University, Changsha 410011, China; 5 Department of Oncology, The Second Xiangya Hospital of Central South University, Changsha 410011, China

**Keywords:** bone tumor, soft tissue sarcomas, multidisciplinary team, diagnosis, treatment, prognosis

## Abstract

Bone and soft tissue sarcomas with complex and varied clinical, imaging, and pathological characteristics cannot be diagnosed and treated by a single discipline, as each discipline has some limitations. This study aimed to explore the role of a multidisciplinary team (MDT) in the diagnosis and treatment of bone and soft tissue sarcomas over the past four consecutive years. The subjects were 269 patients discussed during MDT meetings at a Bone and Soft Tissue Sarcomas Center in South China. The diagnosis, relapse diagnosis, unplanned resection, management of pulmonary nodules, and treatment of refractory and advanced tumors were compared to similar data provided in the literature to (i) determine whether the MDT significantly affected the diagnosis and treatment of bone and soft tissue sarcomas, and (ii) explore trends in the types of patients with bone and soft tissue sarcomas and treatment decision-making since the establishment of the MDT. Results revealed that the MDT significantly improved preoperative diagnostic accuracy for patients with bone and soft tissue sarcomas; the accuracy of diagnosis and relapse diagnosis by the MDT reached 95.42% and 100%, respectively. After an MDT discussion, the positive pathology rate for extended resection after unplanned resection was 81.2%. The overall accuracy of the MDT in determining the nature of pulmonary nodules was 87.1–91.9%. For patients presenting with pulmonary nodules in osteosarcoma, no statistically significant difference in survival was shown between cases discussed by the MDT and those without an MDT discussion (*p* = 0.5751). Collectively, the MDT can play a positive role in accurate preoperative diagnosis, relapse diagnosis, the decision to extend resection after an unplanned resection, and the diagnostic accuracy of pulmonary nodules.

## 1. Introduction

Bone and soft tissue sarcomas are a heterogeneous group of mesenchymal malignancies, including primary or metastatic malignant bone tumors and soft tissue sarcomas [1,2], accounting for approximately 1% of adult and 15% of pediatric malignant tumors [3]. The low incidence and high heterogeneity of imaging and pathological characteristics pose a challenge to the diagnosis and treatment of bone and soft tissue sarcomas. Despite the widespread availability of treatment guidelines, misdiagnosis and improper or negligent surgery still occur frequently, which may largely be attributed to the limited experience within a single discipline. Therefore, the diagnosis and treatment of bone and soft tissue tumors require collaboration between experts from multidisciplinary teams (MDT). Additionally, pulmonary metastasis is common in patients with bone and soft tissue sarcomas. However, not all pulmonary nodules identified on chest computed tomography (CT) are metastatic foci, so the proper management of pulmonary nodules also requires extensive discussion by an MDT. In clinical practice, non-musculoskeletal oncologists may have insufficient knowledge of bone and soft tissue tumors, which could result in frequent misdiagnosis of some sarcomas as benign tumors and lead to inappropriate surgical excision. Thus, MDT discussion is critical for managing bone and soft tissue tumors because it serves not only to guide treatment but also to ensure adherence to the latest clinical guidelines, taking into account the local context. Nonetheless, there is little evidence regarding the value of MDT panel discussions for treating bone and soft tissue sarcoma patients. This study aimed to evaluate the benefit of MDT discussion of these patients in a single Chinese center.

## 2. Methods

### 2.1. The Multidisciplinary Approach

Established in January 2018, the multidisciplinary center at the Second Xiangya Hospital of Central South University is one of the largest bone and soft tissue tumor centers in China that meets international standards. Approximately 300 patients with bone and soft tissue tumors were potential subjects of multidisciplinary discussion during the past four consecutive years. Experts in various specialties are all skilled in the diagnosis and treatment of these tumors.

An MDT panel on the diagnosis and treatment of bone and soft tissue tumors includes orthopedic surgeons, oncologists, radiologists, pathologists, thoracic surgeons, and non-core members, such as clinical pharmacists, nutritionists, psychiatrists, and nurses. Orthopedics is the core of the team, and orthopedic surgeons are mainly responsible for patient consultations regarding bone and soft tissue diseases, hospital admission, preliminary diagnosis, and follow-up. The MDT discussion was held weekly. Outpatients and inpatients submitted their applications for discussion at the MDT center. Prior to the panel discussion, the physicians retrieved the medical history and compiled the imaging data and other relevant information. Information about the patient to be discussed was provided to the MDT experts one day in advance. Generally, the discussion was organized as follows: The medical history of patients, clinical symptoms, physical examination, imaging characteristics, histological findings, and preliminary diagnosis were initially reviewed. Then, the experts who participated in the MDT discussion expressed their views on diagnosis and treatment. Finally, an orthopedic oncologist made a summary, including consensus on the diagnosis, relapse diagnosis, treatment of the disease, features and management of pulmonary nodules, and the potential need for extended resection and unplanned surgery.

### 2.2. Study Population and Design

This study was conducted at the Second Xiangya Hospital of Central South University in Changsha, Hunan Province, China. A total of 269 patients were subjects of MDT discussions from January 2018 (inception) to December 2021. The basic information for all patients, including clinical symptoms, imaging, and pathological findings, was obtained from electronic medical records. MDT discussions concerning these recruited patients were retrospectively examined. This study was approved by the Ethics Committee of Central South University and conducted in accordance with the Declaration of Helsinki. An audit was commenced in January 2018. A total of 331 panel discussions were held regarding 269 patients, as some patients were discussed several times before reaching a final consensus. To reflect the involvement of the MDT in the diagnosis and treatment of bone and soft tissue sarcomas, the period from January 2018 to December 2021 was further divided into four parts.

The 269 patients were sorted into different categories by type of disease. Demographic data were obtained, such as age, gender, tumor site, Enneking stage, theme of discussion, benign or malignant tumors, whether metastasis occurred, and survival. Further data include the accuracy of diagnosis before and after MDT discussion, the accuracy of relapse diagnosis, unplanned excision decisions, and pulmonary nodule management. Tumor treatment effects, namely complete response (CR), partial response (PR), stable disease (SD), or progressive disease (PD), were also assessed. For osteosarcoma patients with pulmonary nodules, we conducted a comparative survival analysis between patients who were discussed by the MDT versus undiscussed patients. The experimental group (*n* = 42) consisted of patients who were discussed by an MDT, and 40 osteosarcoma patients with complete information who did not apply for MDT discussion served as the controls. Multiple parameters, including age, gender, tumor location, Enneking stage, metastasis occurrence, and survival, were compared between the two groups.

### 2.3. Standards and Endpoints

Follow-up began in January 2018. The first postoperative day was used as the initial time point for follow-up. The follow-up schedule was as follows: every three months for years one and two; every four months for year three; every six months for years four and five; and yearly thereafter. Patients with pulmonary nodules had their lung CT reviewed at every follow-up visit and submitted a request for MDT discussion when there were changes in the lung nodules. The starting point for survival analysis was the day of surgery, and the survival analysis endpoint was on 31 December 2021, or upon the patient’s death.

### 2.4. Statistical Analysis

Statistical analysis was performed using the SPSS Statistics 25 package. A *p* < 0.05 was considered to be statistically significant. Categorical variables were expressed as percentages. The rate of age, gender, tumor location, Enneking stage, and metastasis were compared between the experimental and control groups using the chi-square (χ2) test and Fisher’s exact test. Kaplan-Meier estimation was used to create graphs of the observed survival curves, while the log-rank test was used to compare curves from different groups.

## 3. Results

### 3.1. Clinical Characteristics of Patients Discussed by the MDT

**Figure 1:**
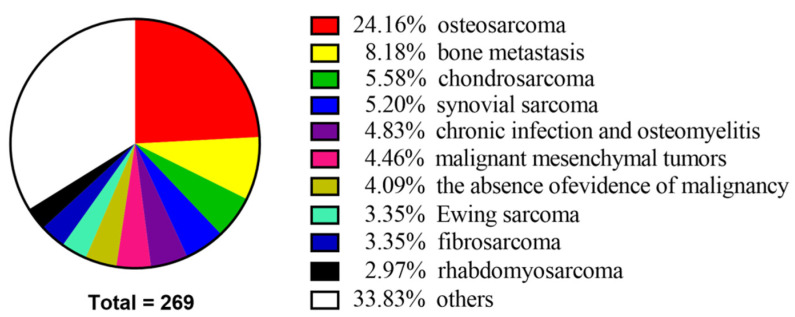
The ten leading causes of MDT patient discussion.

Overall, 269 patients with bone and soft tissue tumors were discussed by the MDT at the Second Xiangya Hospital of Central South University. Among them, 137 (50.9%) were male, and 132 were female (49.1%), with a wide age distribution ranging from 3 to 77 years. The ten leading causes of MDT patient discussion were osteosarcoma (24.16%), bone metastasis (8.18%), chondrosarcoma (5.58%), synovial sarcoma (5.2%), chronic infection and osteomyelitis (4.83%), malignant mesenchymal tumors (4.46%), the absence of evidence of malignancy (4.09%), Ewing sarcoma (3.35%), fibrosarcoma (3.35%), and rhabdomyosarcoma (2.97%) (Figure 1 and Supplementary Materials Table S1). Demographic data on patients discussed by the MDT are shown in Table 1.

**Figure 2:**
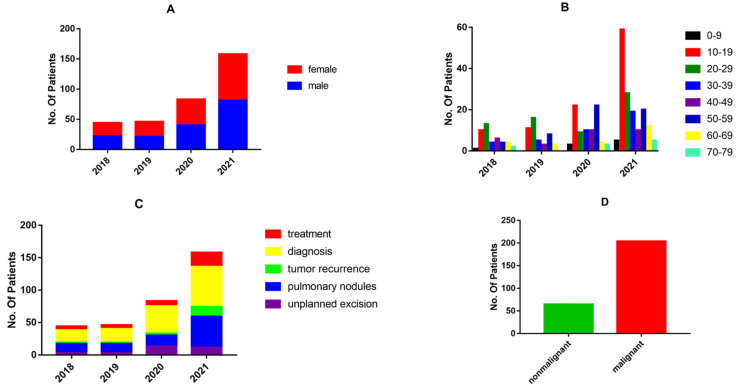
(**A**) The sex distribution of patients discussed by the MDT in 2018–2021; (**B**) The age distribution of patients discussed by the MDT in 2018–2021; (**C**) Topics discussed by the MDT in 2018–2021; (**D**) Distribution of malignant and non-malignant diseases among 269 patients.

To explore the demographic trends and status of bone and soft tissue tumor patients discussed by the MDT, we conducted statistical analyses on a yearly basis from January 2018 to December 2021. Since 47 patients applied for MDT discussion two to four times, a total of 331 discussions were held regarding 269 patients. The following statistics are based on the number of discussions. Of the 44 patients in 2018, 46 patients in 2019, 83 patients in 2020, and 158 patients in 2021, the gender ratio was close to 1 (Figure 2A). The age distribution is demonstrated in Figure 2B. The content discussed at MDT meetings can be broadly divided into five categories: unplanned excision, pulmonary nodules, tumor recurrence, diagnosis, and treatment (Figure 2C). Most MDT discussions (71.6%) were held regarding pulmonary nodules and diagnosis. Of the 269 patients, there were 204 cases (75.8%) of malignant tumors and 65 cases (24.2%) of non-malignant tumors (Figure 2D).

### 3.2. The Role of the MDT in Diagnosis

MDT discussions were held after admission or after a biopsy in 131 patients with an unknown diagnosis, and the diagnosis was corrected in six cases during MDT discussion after a biopsy (Table 2). Representative images of one case are shown in Supplementary Materials S1. Six cases were misdiagnosed by the MDT, of which two cases had clear postoperative pathology in our hospital, and four cases were diagnosed clearly in other hospitals (Table 3). Thus, 125 cases were diagnosed accurately, and six were misdiagnosed, so the accuracy of MDT diagnosis was 95.42%. However, if MDT discussions were not held, the diagnostic accuracy was 90.84%.

### 3.3. The Role of the MDT in Relapse Diagnosis

The MDT discussed the diagnosis of recurrence for a total of 17 cases, of which one case was lost to follow-up, five cases were considered recurrence-free, and 11 cases were considered recurrent (Table 4). The five cases considered recurrence-free had been reviewed for at least six months, and no significant abnormal signal was observed on Magnetic Resonance Imaging (MRI). Among the 11 cases considered as recurrent, eight underwent reoperation, and all were pathologically confirmed as tumor recurrence, and three cases were treated non-operatively and closely followed after review, suggesting tumor progression. Thus, the accuracy rate of relapse diagnosis by the MDT was 100%.

### 3.4. Unplanned Resection

In total, 24 patients underwent unplanned resection and were discussed 30 times. One patient was lost to follow-up. After MDT discussion, non-resection was recommended for six patients, and follow-up and re-examination were conducted. One patient’s follow-up MRI showed an increase in abnormal signal range after one year of close follow-up, and it was decided to perform a wide excision. Wide excision was recommended in 17 cases and rejected in one case. Among the 16 cases of wide excision, three were pathologically negative (18.8%), and 13 were pathologically positive (81.2%). Representative images of one case are shown in Supplementary Materials S2.

### 3.5. Pulmonary Nodules

Pulmonary nodules were discussed by the MDT 93 times for 67 patients with bone and soft tissue sarcomas. Of these, five were lost to follow-up, and 62 were included in the study. At the time of presentation, 29 had no metastases, and 33 had pulmonary metastases; 29 cases presented with pulmonary nodules ranging from 1 to 48 months after consultation.

**Figure 3:**
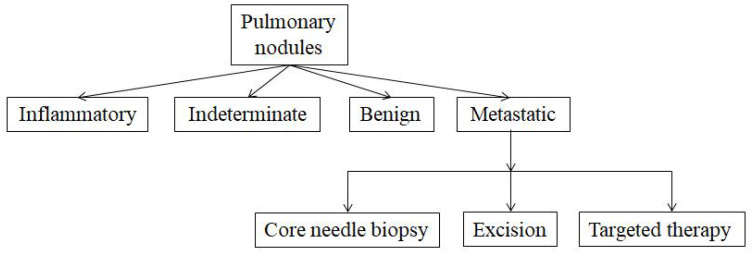
Classification and management pattern after MDT discussion of pulmonary nodules.

There were four pulmonary nodule types: inflammatory, indeterminate, benign, and metastatic nodules (Figure 3). The former three types were usually treated by observation, surveillance, and periodic examination. For metastatic nodules, three management strategies were adopted: core needle biopsy, excision, and targeted therapy. These patients were followed for at least six months (from six months to four years) and underwent at least one chest CT scan.

The MDT considered 24 cases as benign nodules, five cases as inflammatory nodules, four cases as indeterminate nodules, and the remaining 29 cases as metastatic nodules. Among the 24 benign nodules, after multiple reviews over six months, 18 cases were unchanged, five cases disappeared, and one showed progression. While for inflammatory nodules, three cases were unchanged, one disappeared, and one showed progression. All indeterminate pulmonary nodules had no change during the periodic review (Table 5).

Among the 29 cases of metastatic nodules, surgical resection was recommended in 11 cases, and all cases were pathologically confirmed after surgery (Table 6 and representative images of one case are shown in Supplementary Materials S3); a core needle biopsy was recommended in four cases, after which one case was pathologically confirmed, and three cases were negative. Specifically, one of these three negative cases was ultimately diagnosed as a pneumococcal infection. Targeted therapy was recommended in 14 cases. One of which had no change in the lung nodules upon re-examination, 11 cases showed progression, and two cases of pulmonary nodules had disappeared.

Taken together, the diagnostic accuracy of pulmonary nodules in patients with bone and soft tissue sarcomas by the MDT reached 87.1–91.9%.

### 3.6. The Role of the MDT in Treatment

The MDT discussed the treatment of a total of 32 patients with a clear diagnosis of bone and soft tissue malignancies that were either postoperative or metastatic. Among these patients, three cases (9.37%) achieved a CR, two cases (6.25%) achieved a PR, eight cases (25%) experienced SD, and 19 cases (59.38%) had PD. Of the 19 patients in the progressive stage, 15 (78.9%) died (Table 7 and Supplementary Materials Table S2).

### 3.7. Comparison between Groups of Patients with Osteosarcoma Presenting with Pulmonary Nodules Who Were Discussed by the MDT versus Undiscussed Patients and Survival Analysis

**Figure 4:**
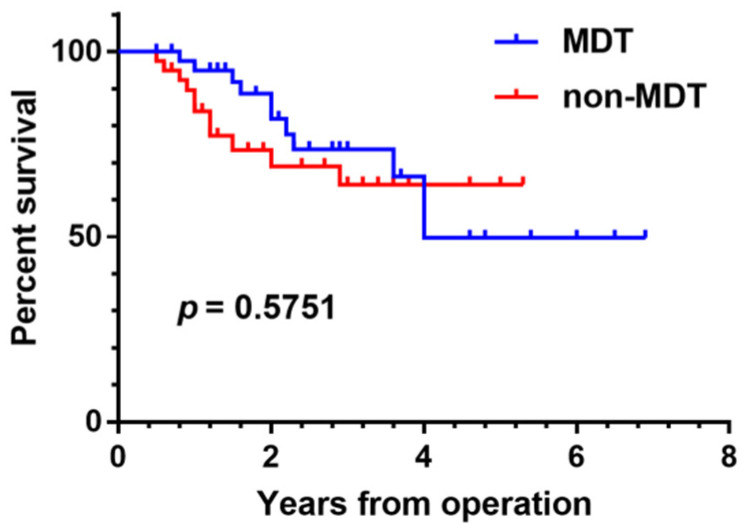
Survival analysis of patients with osteosarcoma who were discussed by the MDT due to pulmonary nodules versus undiscussed patients with osteosarcoma and pulmonary nodules.

The mean survival time was 2.52 years (range 0.5~6.9) and 2.16 years (range 0.5~5) in the MDT and non-MDT groups, respectively. A comparison of the clinical characteristics of patients in the two groups showed a similar median age, gender, tumor location, Enneking stage, metastasis, and survival. There were 21 males and 21 females in the MDT group, aged 19.76 (16, 20) years (range 12–50 years), and 22 males and 18 females in the non-MDT group, aged 16.68 (13, 20.25) years (range 4–52) (Table 8). There was no significant difference in age, gender, tumor location, Enneking stage, metastasis, and survival between groups (*p* > 0.05). For osteosarcoma patients presenting with pulmonary nodules, the 3-year overall survival (OS) rate in the MDT group and the non-MDT group was 75% and 60.7%, respectively, and the 5-year progression-free survival (PFS) rate in the two groups was 79.3% and 58.6%, respectively. For osteosarcoma patients presenting with pulmonary nodules, no statistically significant difference in survival was found between the MDT and non-MDT groups (*p* = 0.5751) (Figure 4). 

## 4. Discussion

Bone and soft tissue sarcomas are a highly heterogeneous group of mesenchymal malignancies, including malignant bone tumors [4]. Primary malignant bone tumors are extremely rare neoplasms accounting for fewer than 0.2% of all malignancies, although the actual incidence is difficult to determine [5]. Soft tissue sarcomas contribute to 1% of all adult malignancies, with more than 100 histologic subtypes that have developed from or within muscle, fat, nerve, cartilage, and bone tissues [6]. The low incidence of bone and soft tissue tumors is further complicated by their variable histological, imaging, and clinical manifestations. The same type of tumor may have different radiographic characteristics, and different tumors may show similar imaging features. The early clinical features of bone and soft tissue tumors are generally not obvious. Even if bone and soft tissue tumors have typical imaging features, physicians can only make a preliminary diagnosis based on imaging results, and pathological examination is required to establish the final diagnosis. Histological examination is usually considered the most accurate diagnostic method, but these pathologic observations are only one of the diagnostic criteria for bone and soft tissue tumors. Pathologic tissue biopsy may also be affected by many factors, such as the biopsy site and preservation of specimens, which may bring certain difficulties to pathological diagnosis and increase the misdiagnosis rate. Accordingly, the diagnosis and treatment of bone and soft tissue tumors cannot be made by one discipline alone. Therefore, a multidisciplinary collaboration among orthopedic surgeons, radiologists, pathologists, oncologists, thoracic surgeons, and other specialists is required to reduce the rate of misdiagnosis, underdiagnosis, and mistreatment so that patients can receive standardized and precise treatment.

To the best of our knowledge, this is the first study to investigate the role of an MDT in the management of diagnosis and treatment of bone and soft tissue tumors.

Diagnosis and pulmonary nodules were the main topics of the MDT discussions. The number of cases discussed by the MDT increased every year, with the number of cases in 2021 being almost twice as high as in 2020. This indicates that MDT discussions are becoming increasingly widespread in the diagnosis and treatment of bone and soft tissue tumors.

In recent studies, the diagnostic accuracy of bone and soft tissue tumors based on core needle biopsies ranged from 74% to 98% [7,8,9,10,11]. In line with these results, we identified an overall diagnostic accuracy of 90.84% in bone and soft tissue tumors without MDT discussion. However, the MDT discussion increased the diagnostic accuracy to 95.42%. Diagnosis of bone and soft tissue tumors based on imaging and pathological observations is complex and difficult. The pathological tissue subtypes are diverse and have overlapping histological features. The advantage of the MDT discussions in improving diagnostic accuracy is evident. For certain difficult cases, through preoperative MDT discussions, radiologists and pathologists can communicate with orthopedic surgeons and advise the most suitable site for biopsy, which may reduce the probability of invalid specimens and improve the quality of specimens. For cases in which the pathological diagnosis is still unclear after biopsy, an MDT discussion can clarify whether to perform a repeat biopsy, switch from core-needle to open biopsy, or proceed to the next step of treatment, which improves the efficiency of diagnosis and the quality of treatment.

Despite effective treatment, local recurrence or distant metastases of bone and soft tissue sarcomas occur in up to 60% of patients who initially receive treatment [12,13]. Recurrent or refractory bone and soft tissue sarcomas have a very poor prognosis in children. Second-line chemotherapy regimens or newly developed agents have not improved the outcome. OS rates have been reported to be less than 40% after relapse in most series [14,15,16,17,18,19,20,21,22]. Survival rates for osteosarcoma and Ewing sarcoma after relapse are less than 30% [17,19,23] and 23% [17,22], respectively. Soft tissue sarcoma local recurrences occur in approximately 10% of patients treated with margin-negative resection. Most recurrences occur within the first two years [24]. Given the high risk of recurrence in this patient population, the accurate and early detection of local or recurrent metastatic lesions is highly warranted because early treatment can prolong survival. The diagnostic sensitivity and specificity using contrast-enhanced CT alone were 78% and 67%, respectively, with an accuracy of 73%. In contrast, the diagnostic sensitivity and specificity of ^18^F-FDG PET/CT were 94% and 92%, respectively, with an accuracy of 93%. ^18^F-FDG PET/CT was particularly superior regarding the detection of local recurrence of soft tissue lesions (sensitivity and specificity: 83% and 100% vs. 50% and 100%, respectively) or bone metastases (100% and 100% vs. 85% and 88%, respectively) [25]. In our study, 16 patients were included in the discussion of a recurrence diagnosis. Of the 11 patients considered for recurrence after MDT discussion, eight cases underwent reoperation, and all had positive postoperative pathology. The three patients who did not undergo reoperation had worsening lesions upon re-examination. In the five cases not considered as recurrence, no significant imaging changes were observed upon re-examination. The accuracy of relapse diagnosis by the MDT reached 100%, and the MDT improved the accuracy of a recurrence diagnosis of bone and soft tissue tumors compared to the available literature.

Unplanned resection usually refers to inappropriate surgical resection of a soft tissue sarcoma misdiagnosed as a benign tumor, resulting in a positive tumor specimen margin or residual tumor. Unplanned resection, which continues to be a major issue for musculoskeletal oncologists, is common in bone and soft tissue sarcomas. Osseous malignancies with a non-classic presentation are frequently misdiagnosed; therefore, they receive inappropriate surgical management. Those referred for secondary treatment after an unplanned resection are more likely to have a sarcoma with a diameter of <5 cm that is superficially located and painless [26,27,28,29]. Lesions with the above manifestations are regarded as benign tumors. In addition, there is currently no effective referral system in China. Patients with bone and soft tissue tumors, especially soft tissue tumors, are treated in orthopedics, general surgery, and even other departments. Even when treatment is provided in orthopedic departments, it is often not administered by an orthopedic oncologist. In addition, up to one-third of all soft tissue sarcomas arise superficially to the investing muscular fascia, and they can range in size from a few millimeters to several tens of centimeters [27]. Reported rates of residual sarcoma in the re-resected specimen after unplanned resections are uniformly high, ranging from 24% to 74% [26,28,30,31,32,33,34,35]. In contrast, the residual rate of re-excision after MDT discussion at our center was 81.2%, higher than that reported in the literature. These data may indicate to some extent that the MDT plays an active role in the decision to perform re-excision after unplanned resection.

The lung is the most common site for sarcoma metastasis. It is reported that approximately 15–20% of osteosarcoma patients suffered from distant metastases at the time of presentation, with more than 85% of these metastases being pulmonary [36]. Of patients with extremity sarcomas, approximately 20% may proceed with isolated pulmonary metastatic disease at some point in the course of their disease [37]. Since CT examination is more sensitive than chest plain film, it has been regarded as the best method to diagnose metastatic pulmonary tumors. However, there are many studies showing that CT cannot detect the exact number of pulmonary metastases accurately [38,39]. It is well recognized that not all pulmonary nodules identified on a chest CT in patients with sarcoma represent metastases [40,41] and that a chest CT is relatively insensitive compared to lung palpation/thoracotomy in the identification of pulmonary metastases [42,43,44]. CT has become the standard for detecting and monitoring pulmonary lesions, but it frequently identifies nodules of uncertain clinical significance. Often the assumption is that, especially in children, these nodules represent metastatic disease. However, up to 60% of pulmonary nodules in adults and 33% in children may be non-malignant [45,46].

Differentiating benign and malignant lesions is essential for planning treatment and determining prognosis. When nodules are detected, invasive procedures may be necessary to establish the histopathologic diagnosis, but in many cases, the nodules are simply monitored with periodic CT scans. From the patients’ perspective, these nodules may cause psychological distress due to the uncertain origin and biological features of the nodules. Therefore, the diagnosis of pulmonary nodules requires multidisciplinary cooperation so that the strengths of each department can be better utilized to offer patients individualized treatment. The pulmonary nodules were broadly classified into four types: benign, metastatic, indeterminate, and inflammatory. After the MDT discussion, inflammatory, benign, and indeterminate pulmonary nodules were monitored with CT scans. In our MDT, the accuracy of diagnosis of benign pulmonary nodules achieved 95.8%. Of the 24 patients, only one showed progressive lesions after review, five showed a disappearance of pulmonary nodules, and 18 showed no significant changes. Four cases of indeterminate pulmonary nodules without significant changes on multiple re-examinations were reported. Among the five cases of inflammatory nodules, one case showed progressive lesions, three cases showed no significant changes, and one case of pulmonary nodules disappeared (Table 5). The 29 cases considered to be metastatic pulmonary nodules were managed in three ways, metastasectomy, biopsy, and targeted therapy, according to the condition and resectability of the pulmonary nodules. We found that postoperative pathology was positive in all pulmonary nodules undergoing metastasectomy, while in three of the four cases for which the MDT recommended puncture biopsy, postoperative pathology was negative. Of the 14 patients for whom targeted therapy was recommended by the MDT, 11 cases had progressive lung nodule lesions, nodules disappeared in two cases, and one case had no change (Table 6). The possible causes of the three pulmonary nodules without progressive lesions were either effective treatment or incorrect MDT judgement. Therefore, the overall accuracy of the MDT in determining the nature of pulmonary nodules was 87.1–91.9%. In contrast, the literature reports that between 68% and 74% of nodules were correctly classified by the reporting CT radiologists [47]. This suggests that an MDT can improve the diagnostic accuracy of pulmonary nodules and better guide treatment.

Indeterminate pulmonary nodules present a continued diagnostic dilemma for a large range of solid cancers, and there is a clear paucity in the literature guiding best practices in their management [48,49]. Regarding the need for a multidisciplinary approach in the treatment of rare diseases such as metastatic soft tissue sarcomas [50], cooperation between orthopedic surgeons, pathologists, oncologists, and thoracic surgeons is of utmost importance. In the current literature, the resection of pulmonary nodules remains a vital cornerstone in treating patients with bone and soft tissue sarcoma metastasis. The thoracic surgeon has therefore become an integral part of the sarcoma team. The accurate diagnosis of pulmonary metastases, specifically differentiating them from infection or non-specific pulmonary nodules, requires close cooperation with oncologists and radiologists.

We conducted a comparative survival analysis between groups of patients with osteosarcoma presenting with pulmonary nodules who were discussed by the MDT versus undiscussed patients. There was no significant difference in age, gender, tumor location, Enneking stage, metastasis, and survival between the two groups (*p* > 0.05). Our study did not demonstrate any statistically significant survival benefit from MDT management of osteosarcoma patients with pulmonary nodules (*p* = 0.5751) (Figure 4). However, in the first four years of follow-up, it can be seen that the survival time curve for patients who were subjects of the MDT discussions is superior to that of undiscussed patients. It remains to be further validated if early survival rates are higher in patients discussed by the MDT. Possible explanations for the absence of any significant survival difference between the two groups include the insufficient number of cases, the comparatively short follow-up time, and the potential bias of selected cases. 

Despite significant improvements in modern multimodality treatment, the outcomes and OS rates remain poor for bone and soft tissue sarcoma patients with advanced, refractory, metastatic, or relapsed lesions. The prognosis of those patients has risen from one year in the 1980s and 1990s to 18 months in the last two decades [51,52]. The MDT discussed the treatment of a total of 32 patients with a clear diagnosis of bone and soft tissue malignancies that were either postoperative or metastatic. Among these patients, three cases (9.37%) obtained a CR, two cases (6.25%) achieved a PR, eight cases (25%) experienced SD, and 19 cases (59.38%) had progressive disease. Of the 19 patients in the progressive stage, 15 (78.9%) died. Most of the patients discussed by the MDT for treatment were those with multiple metastases, pelvic malignancies, and tumors of high malignancy; therefore, the prognosis was poor. Those patients with CR, such as isolated fibrous tumors, bone metastases from breast cancer, and chondrosarcoma, are less malignant or have a better prognosis. All but one of the patients in the progressive stage died within two years, and the majority died within one year, which is generally consistent with what has been reported in the literature [51,52,53,54,55]. An MDT may not significantly effect survival in advanced bone and soft tissue tumors.

Our study has several limitations, including the inherent limitations of a unicentric and retrospective study. Moreover, the MDT was not blinded and had access to the initial clinical plan, which may have introduced potential bias. Additionally, in our study, the follow-up time was not very long, and some cases were excluded.

In conclusion, the rarity of bone and soft tissue sarcomas and the difficulty in interpreting the imaging and histology results make diagnosis and treatment difficult and complex. Precise and standardized diagnosis and treatment require systematic MDT management of patients with these diseases. MDT discussion can play an important role in the diagnosis and treatment of bone and soft tissue tumors.

## Figures and Tables

**Table 1:** Demographic data of patients discussed by the MDT during each year of the study.

Patient Characteristics	2018	2019	2020	2021
Sex				
Male	22 (50.0)	21 (45.7)	40 (48.2)	81 (51.3)
Female	22 (50.0)	25 (54.3)	43 (51.8)	77 (48.7)
Age of Onset				
0–9	1 (2.3)	0 (0)	3 (3.6)	5 (3.2)
10–19	10 (22.7)	11 (23.9)	22 (26.5)	59 (37.3)
20–29	13 (29.6)	16 (34.8)	9 (10.8)	28 (17.7)
30–39	4 (9.1)	5 (10.9)	10 (12.1)	19 (12.0)
40–49	6 (13.6)	3 (6.5)	10 (12.1)	10 (6.3)
50–59	4 (9.1)	8 (17.4)	22 (26.5)	20 (12.7)
60–69	4 (9.1)	3 (6.5)	4 (4.8)	12 (7.6)
70–79	2 (4.6)	0 (0)	3 (3.6)	5 (3.2)
Theme of Discussion				
Unplanned Excision	3 (6.8)	3 (6.5)	13 (15.7)	11 (7.0)
Pulmonary Nodules	14 (31.8)	14 (30.4)	17 (20.5)	48 (30.4)
Tumor Recurrence	2 (4.6)	2 (4.4)	3 (3.6)	15 (9.5)
Diagnosis	19 (43.2)	21 (45.7)	42 (50.6)	62 (39.2)
Treatment	6 (13.6)	6 (13.0)	8 (9.6)	22 (13.9)

**Table 2:** The diagnosis corrected in six cases during MDT discussion after a biopsy.

Sex	Age	Biopsy Diagnosis	MDT Time	MDT Diagnosis
M	30	Osteosarcoma	Post-biopsy	Myositis ossificans
M	45	Low-grade malignant mesenchymal tumor	Post-biopsy	Giant cell tumor of bone
F	17	Fibrosarcoma	Post-biopsy	Osteosarcoma
M	27	Aggressive fibromatosis	Post-biopsy	Deep fibrous histiocytoma
F	31	Angiosarcoma	Post-biopsy	Aggressive angiomyxoma
F	3	Osteosarcoma	Post-biopsy	Bone infection

**Table 3:** Six misdiagnosed cases by the MDT.

Sex	Age	Biopsy Diagnosis	MDT Time	MDT Diagnosis	Final Diagnosis
F	22	Low-grade malignant mesenchymal neoplasm	Post-biopsy	Myositis ossificans	Parosteal osteosarcoma
M	13	The absence of evidence of malignancy	Post-biopsy	Invasive osteoblastoma	Well-differentiated osteosarcoma
F	52	NA	Pre-operation	Giant cell tumor of bone	Osteosarcoma
M	9	Low-grade osteosarcoma	Post-biopsy	Malignant bone tumor	Periosteal fasciitis
F	50	Inflammatory cell infiltration	Post-operation	Cystosarcoma phyllodes	Extraskeletal osteosarcoma
F	19	The absence of evidence of malignancy	Post-biopsy	Bone infection	Bone metastasis of lung cancer

**Table 4:** Management and outcome of 16 cases which the MDT considered as relapse diagnosis.

Recurrence				Non-Recurrence	
Resection		Non-Surgical Treatment		No Abnormal Signal on MRI	Abnormal Signal on MRI
Positive	Negative	Deterioration	No Deterioration		
8	0	3	0	5	0

**Table 5:** Management and outcome of non-metastatic pulmonary nodules.

	Benign Pulmonary Nodules	Inflammatory Pulmonary Nodules	Indeterminate Pulmonary Nodules
Unchanged	18	3	4
Disappeared	5	1	0
Progressive	1	1	0
Total	24	5	4
Accuracy	95.8%	80%	100%

**Table 6:** Management and outcome of metastatic pulmonary nodules.

	Excision	Core Needle Biopsy	Targeted Therapy
Pathologically Positive	11	1	
Pathologically Negative	0	3	
Unchanged			1
Disappeared			2
Progressive			11
Total	11	4	14
Accuracy	100%	25%	78.6–100%

**Table 7:** Outcome of 32 patients after MDT discussion of treatment plan.

Therapeutic Evaluation	No. of Patients	Percentage
Complete Response (CR)	3	9.37%
Partial Response (PR)	2	6.25%
Stable Disease (SD)	8	25%
Progressive Disease (PD)	19	59.38%

**Table 8:** Patient characteristics.

Characteristics	MDT Group	Non-MDT Group	*p* Value
Age	19.76 (16–20)	16.68 (12–19.25)	0.086
Gender			0.65
Male	21	22	
Female	21	18	
Tumor Location			0.811
Femur	21	18	
Tibia	14	12	
Humerus	3	7	
Radius	1	1	
Fibula	1	1	
Pelvis	2	1	
Enneking Stage			0.49
IA	0	0	
IB	0	0	
IIA	0	1	
IIB	31	31	
III	11	8	
Metastasis			0.507
Yes	11	8	
No	31	32	
Survival Status			0.341
Alive	31	19	
Dead	11	11	

## Data Availability

Not applicable.

## Supplementary Materials

The following supporting information can be downloaded at: https://www.mdpi.com/article/10.3390/jpm12122079/s1, File S1: Appendix. MDT Center-Case Management. This supplementary file demonstrates an example of case management carried out by an MDT specialized in the diagnosis and treatment of bone and soft tissue tumor. Table S1. Disease classification and summary of MDT patients. S1. Representative images of which the diagnosis was corrected in one case. S2. Representative images of lesion pathologically confirmed after wide excision in patient undergoing unplanned resection. S3. Representative images of metastatic nodules pathologically confirmed after surgery in patients with osteosarcoma. Table S2. Demographic data and outcome of 32 patients after MDT discussion for treatment plan.

## Abbreviations

MDT	multidisciplinary team
CT	computed tomography
CR	complete response
PR	partial response
SD	stable disease
PD	progressive disease
MRI	magnetic resonance imaging
OS	overall survival
PFS	progression-free survival

## References

[1] Callegaro D., Miceli R., Mariani L., Raut C.P., Gronchi A. (2017). Soft tissue sarcoma nomograms and their incorporation into practice. Cancer.

[2] Thoenen E., Curl A., Iwakuma T. (2019). TP53 in bone and soft tissue sarcomas. Pharmacol. Ther..

[3] Miwa S., Yamamoto N., Hayashi K., Takeuchi A., Igarashi K., Tsuchiya H. (2019). Therapeutic Targets for Bone and Soft-Tissue Sarcomas. Int. J. Mol. Sci..

[4] Gatta G., van der Zwan J.M., Casali P.G., Siesling S., Dei Tos A.P., Kunkler I., Otter R., Licitra L., Mallone S., Tavilla A. (2011). Rare cancers are not so rare: The rare cancer burden in Europe. Eur. J. Cancer.

[5] Biermann J.S., Adkins D.R., Agulnik M., Benjamin R.S., Brigman B., Butrynski J.E., Cheong D., Chow W., Curry W.T., Frassica D.A. (2013). Bone cancer. J. Natl. Compr. Canc. Netw..

[6] Gamboa A.C., Gronchi A., Cardona K. (2020). Soft-tissue sarcoma in adults: An update on the current state of histiotype-specific management in an era of personalized medicine. CA Cancer J. Clin..

[7] Issakov J., Flusser G., Kollender Y., Merimsky O., Lifschitz-Mercer B., Meller I. (2003). Computed tomography-guided core needle biopsy for bone and soft tissue tumors. Isr. Med. Assoc. J..

[8] Mitsuyoshi G., Naito N., Kawai A., Kunisada T., Yoshida A., Yanai H., Dendo S., Yoshino T., Kanazawa S., Ozaki T. (2006). Accurate diagnosis of musculoskeletal lesions by core needle biopsy. J. Surg. Oncol..

[9] Altuntas A.O., Slavin J., Smith P.J., Schlict S.M., Powell G.J., Ngan S., Toner G., Choong P.F. (2005). Accuracy of computed tomography guided core needle biopsy of musculoskeletal tumours. ANZ J. Surg..

[10] Torriani M., Etchebehere M., Amstalden E. (2002). Sonographically guided core needle biopsy of bone and soft tissue tumors. J. Ultrasound Med..

[11] Welker J.A. (2000). The percutaneous needle biopsy is safe and recommended in the diagnosis of musculoskeletal masses. Cancer.

[12] Johnson G.R., Zhuang H., Khan J., Chiang S.B., Alavi A. (2003). Roles of positron emission tomography with fluorine-18-deoxyglucose in the detection of local recurrent and distant metastatic sarcoma. Clin. Nucl. Med..

[13] Pisters P.W., Leung D.H., Woodruff J., Shi W., Brennan M.F. (1996). Analysis of prognostic factors in 1041 patients with localized soft tissue sarcomas of the extremities. J. Clin. Oncol..

[14] Pappo A.S. (1999). Survival After Relapse in Children and Adolescents With Rhabdomyosarcoma: A Report From the Intergroup Rhabdomyosarcoma Study Group. J. Clin. Oncol..

[15] Mazzoleni S., Bisogno G., Garaventa A., Cecchetto G., Ferrari A., Sotti G., Donfrancesco A., Madon E., Casula L., Carli M. (2005). Outcomes and prognostic factors after recurrence in children and adolescents with nonmetastatic rhabdomyosarcoma. Cancer.

[16] Winter S., Fasola S., Brisse H., Mosseri V., Orbach D. (2015). Relapse after localized rhabdomyosarcoma: Evaluation of the efficacy of second-line chemotherapy. Pediatr. Blood Cancer.

[17] Miser J.S., Kinsella T.J., Triche T.J., Tsokos M., Jarosinski P., Forquer R., Wesley R., Magrath I. (1987). Ifosfamide with mesna uroprotection and etoposide: An effective regimen in the treatment of recurrent sarcomas and other tumors of children and young adults. J. Clin. Oncol..

[18] Grier H.E. (2003). Addition of Ifosfamide and Etoposide to Standard Chemotherapy for Ewing’s Sarcoma and Primitive Neuroectodermal Tumor of Bone. New Engl. J. Med..

[19] Hawkins D.S., Arndt C.A. (2003). Pattern of disease recurrence and prognostic factors in patients with osteosarcoma treated with contemporary chemotherapy. Cancer.

[20] Gelderblom H., Jinks R.C., Sydes M., Bramwell V.H., van Glabbeke M., Grimer R.J., Hogendoorn P.C., McTiernan A., Lewis I.J., Nooij M.A. (2011). Survival after recurrent osteosarcoma: Data from 3 European Osteosarcoma Intergroup (EOI) randomized controlled trials. Eur. J. Cancer.

[21] Leavey P.J., Mascarenhas L., Marina N., Chen Z., Krailo M., Miser J., Brown K., Tarbell N., Bernstein M.L., Granowetter L. (2008). Prognostic factors for patients with Ewing sarcoma (EWS) at first recurrence following multi-modality therapy: A report from the Children’s Oncology Group. Pediatr. Blood Cancer.

[22] Stahl M., Ranft A., Paulussen M., Bolling T., Vieth V., Bielack S., Gortitz I., Braun-Munzinger G., Hardes J., Jurgens H. (2011). Risk of recurrence and survival after relapse in patients with Ewing sarcoma. Pediatr. Blood Cancer.

[23] Kung F.H., Pratt C.B., Vega R.A., Jaffe N., Strother D., Schwenn M., Nitschke R., Homans A.C., Holbrook C.T., Golembe B. (1993). Ifosfamide/etoposide combination in the treatment of recurrent malignant solid tumors of childhood. A Pediatric Oncology Group Phase II study. Cancer.

[24] Crompton J.G., Ogura K., Bernthal N.M., Kawai A., Eilber F.C. (2018). Local Control of Soft Tissue and Bone Sarcomas. J. Clin. Oncol..

[25] Al-Ibraheem A., Buck A.K., Benz M.R., Rudert M., Beer A.J., Mansour A., Pomykala K.L., Haller B., Juenger H., Scheidhauer K. (2013). (18) F-fluorodeoxyglucose positron emission tomography/computed tomography for the detection of recurrent bone and soft tissue sarcoma. Cancer.

[26] Fiore M., Casali P.G., Miceli R., Mariani L., Bertulli R., Lozza L., Collini P., Olmi P., Mussi C., Gronchi A. (2006). Prognostic effect of re-excision in adult soft tissue sarcoma of the extremity. Ann. Surg. Oncol..

[27] Rougraff B.T., Davis K., Cudahy T. (2005). The impact of previous surgical manipulation of subcutaneous sarcoma on oncologic outcome. Clin. Orthop. Relat. Res..

[28] Potter B.K., Adams S.C., Pitcher J.D., Temple H.T. (2008). Local recurrence of disease after unplanned excisions of high-grade soft tissue sarcomas. Clin. Orthop. Relat. Res..

[29] Hoshi M., Ieguchi M., Takami M., Aono M., Taguchi S., Kuroda T., Takaoka K. (2008). Clinical problems after initial unplanned resection of sarcoma. Jpn. J. Clin. Oncol..

[30] Venkatesan M., Richards C.J., McCulloch T.A., Perks A.G., Raurell A., Ashford R.U. (2012). East Midlands Sarcoma, S. Inadvertent surgical resection of soft tissue sarcomas. Eur. J. Surg. Oncol..

[31] Lewis J.J., Leung D., Espat J., Woodruff J.M., Brennan M.F. (2000). Effect of reresection in extremity soft tissue sarcoma. Ann. Surg..

[32] Sugiura H., Takahashi M., Katagiri H., Nishida Y., Nakashima H., Yonekawa M., Iwata H. (2002). Additional wide resection of malignant soft tissue tumors. Clin. Orthop. Relat. Res..

[33] Lin P.P., Guzel V.B., Pisters P.W., Zagars G.K., Weber K.L., Feig B.W., Pollock R.E., Yasko A.W. (2002). Surgical management of soft tissue sarcomas of the hand and foot. Cancer.

[34] Manoso M.W., Frassica D.A., Deune E.G., Frassica F.J. (2005). Outcomes of re-excision after unplanned excisions of soft-tissue sarcomas. J. Surg. Oncol..

[35] Chandrasekar C.R., Wafa H., Grimer R.J., Carter S.R., Tillman R.M., Abudu A. (2008). The effect of an unplanned excision of a soft-tissue sarcoma on prognosis. J. Bone Joint Surg. Br..

[36] Bielack S.S., Kempf-Bielack B., Delling G., Exner G.U., Flege S., Helmke K., Kotz R., Salzer-Kuntschik M., Werner M., Winkelmann W. (2002). Prognostic factors in high-grade osteosarcoma of the extremities or trunk: An analysis of 1702 patients treated on neoadjuvant cooperative osteosarcoma study group protocols. J. Clin. Oncol..

[37] Gadd M.A., Casper E.S., Woodruff J.M., McCormack P.M., Brennan M.F. (1993). Development and treatment of pulmonary metastases in adult patients with extremity soft tissue sarcoma. Ann. Surg..

[38] Ciccarese F., Bazzocchi A., Ciminari R., Righi A., Rocca M., Rimondi E., Picci P., Bacchi Reggiani M.L., Albisinni U., Zompatori M. (2015). The many faces of pulmonary metastases of osteosarcoma: Retrospective study on 283 lesions submitted to surgery. Eur. J. Radiol..

[39] Ellis M.C., Hessman C.J., Weerasinghe R., Schipper P.H., Vetto J.T. (2011). Comparison of pulmonary nodule detection rates between preoperative CT imaging and intraoperative lung palpation. Am. J. Surg..

[40] Khokhar S., Vickers A., Moore M.S., Mironov S., Stover D.E., Feinstein M.B. (2006). Significance of non-calcified pulmonary nodules in patients with extrapulmonary cancers. Thorax.

[41] Caparica R. (2016). Pulmonary Nodules in Patients With Nonpulmonary Cancer: Not Always Metastases. J. Glob. Oncol..

[42] Kayton M.L., Huvos A.G., Casher J., Abramson S.J., Rosen N.S., Wexler L.H., Meyers P., LaQuaglia M.P. (2006). Computed tomographic scan of the chest underestimates the number of metastatic lesions in osteosarcoma. J. Pediatr. Surg..

[43] Heaton T.E., Hammond W.J., Farber B.A., Pallos V., Meyers P.A., Chou A.J., Price A.P., LaQuaglia M.P. (2017). A 20-year retrospective analysis of CT-based pre-operative identification of pulmonary metastases in patients with osteosarcoma: A single-center review. J. Pediatr. Surg..

[44] Gao E., Li Y., Zhao W., Zhao T., Guo X., He W., Wu W., Zhao Y., Yang Y. (2019). Necessity of thoracotomy in pulmonary metastasis of osteosarcoma. J. Thorac. Dis..

[45] Bearcroft P.W., Davies A.M. (1999). Follow-up of musculoskeletal tumours. 2. Metastatic disease. Eur. Radiol..

[46] Picci P., Vanel D., Briccoli A., Talle K., Haakenaasen U., Malaguti C., Monti C., Ferrari C., Bacci G., Saeter G. (2001). Computed tomography of pulmonary metastases from osteosarcoma: The less poor technique. A study of 51 patients with histological correlation. Ann. Oncol..

[47] Brader P., Abramson S.J., Price A.P., Ishill N.M., Emily Z.C., Moskowitz C.S., La Quaglia M.P., Ginsberg M.S. (2011). Do characteristics of pulmonary nodules on computed tomography in children with known osteosarcoma help distinguish whether the nodules are malignant or benign?. J. Pediatr. Surg..

[48] Saifuddin A., Baig M.S., Dalal P., Strauss S.J. (2021). The diagnosis of pulmonary metastases on chest computed tomography in primary bone sarcoma and musculoskeletal soft tissue sarcoma. Br. J. Radiol..

[49] Tsoi K.M., Lowe M., Tsuda Y., Lex J.R., Fujiwara T., Almeer G., Gregory J., Stevenson J., Evans S.E., Botchu R. (2021). How Are Indeterminate Pulmonary Nodules at Diagnosis Associated with Survival in Patients with High-Grade Osteosarcoma?. Clin. Orthop. Relat. Res..

[50] Gronchi A., Miah A.B., Dei Tos A.P., Abecassis N., Bajpai J., Bauer S., Biagini R., Bielack S., Blay J.Y., Bolle S. (2021). Soft tissue and visceral sarcomas: ESMO-EURACAN-GENTURIS Clinical Practice Guidelines for diagnosis, treatment and follow-up(). Ann. Oncol..

[51] Italiano A., Mathoulin-Pelissier S., Cesne A.L., Terrier P., Bonvalot S., Collin F., Michels J.J., Blay J.Y., Coindre J.M., Bui B. (2011). Trends in survival for patients with metastatic soft-tissue sarcoma. Cancer.

[52] Nagar S.P., Mytelka D.S., Candrilli S.D., D’Yachkova Y., Lorenzo M., Kasper B., Lopez-Martin J.A., Kaye J.A. (2018). Treatment Patterns and Survival among Adult Patients with Advanced Soft Tissue Sarcoma: A Retrospective Medical Record Review in the United Kingdom, Spain, Germany, and France. Sarcoma.

[53] McTiernan A., Driver D., Michelagnoli M.P., Kilby A.M., Whelan J.S. (2006). High dose chemotherapy with bone marrow or peripheral stem cell rescue is an effective treatment option for patients with relapsed or progressive Ewing’s sarcoma family of tumours. Ann. Oncol..

[54] Leary S.E., Wozniak A.W., Billups C.A., Wu J., McPherson V., Neel M.D., Rao B.N., Daw N.C. (2013). Survival of pediatric patients after relapsed osteosarcoma: The St. Jude Children’s Research Hospital experience. Cancer.

[55] Lochner J., Menge F., Vassos N., Hohenberger P., Kasper B. (2020). Prognosis of Patients with Metastatic Soft Tissue Sarcoma: Advances in Recent Years. Oncol. Res. Treat..

