# Supplementary material for: The Role of a Multidisciplinary Team in the Diagnosis and Treatment of Bone and Soft Tissue Sarcomas: A Single-Center Experience
Source: J Pers Med. 2022 Dec 16;12(12):2079. doi: 10.3390/jpm12122079 (PMC9782466; doi:10.3390/jpm12122079)
Supplement: Supplementary file 1 [file jpm-12-02079-s001.zip › File S1. APPENDIX.pdf]

APPENDIX

MDT case demonstration

Case summary: Patient was admitted with a chief complaint of “pain in the left knee for more than 1 month.” The patient complained of pain with no apparent cause since July 20, 2020, which was paroxysmal in nature and gradually worsened to the point of inability to walk, accompanied by fever (maximum temperature 37.5°C). Treatment at the local hospital did not improve symptoms. MRI of the knee joint was performed, and inflammation or infection was considered a possibility. After anti-infection treatment, the left knee pain was relieved more than before. In order to seek further diagnosis and treatment, he went to our outpatient clinic. Enhanced MRI examination of the left femur showed intra-medullary lesions in the lower femur bilaterally, with the left side being obvious and involving the periosteum, considering the possibility of bone infarction, with the left side having a larger lesion, not excluding infection.

Current diagnosis: multiple bone destruction.

Purpose of the consultation: diagnosis? Next steps?

Radiologist: X-ray showed moth-eaten destruction of the lower left femoral epiphysis with laminar periosteal reaction. CT further showed bone destruction with laminar periosteal reaction. MRI showed loss of fatty signal in the bone marrow cavity, a low T1 signal, a bilinear sign of T2 signal, and an enhanced signal in soft tissue next to the left lower femoral segment, considering bone infarction.

PET-CT specialist: Imaging suggests laminar thickening of the periosteum and bone destruction unlike the signs of bone tumor, so the considered diagnosis is low toxicity infection.

Orthopedic oncologist: (1) Hematopoietic system examination needs to be performed. (2) Osteomyelitis is less likely, with infection starting from the epiphysis and less from the bone cortex, and osteomyelitis on both sides at the same time is less likely. (3) Bone tumors need to be excluded, especially Ewing sarcoma with laminar periosteal reaction and large lesions.

Preliminary diagnosis:

1. Multiple bone destruction caused by hematological disorders.

2. Malignant bone tumor to be excluded.

Next step: Biopsy on hold and bone marrow aspiration is performed first.

Figure A1. A multidisciplinary team (MDT) for Bone and soft tissue sarcomas was established at this hospital in Janurary 2018.

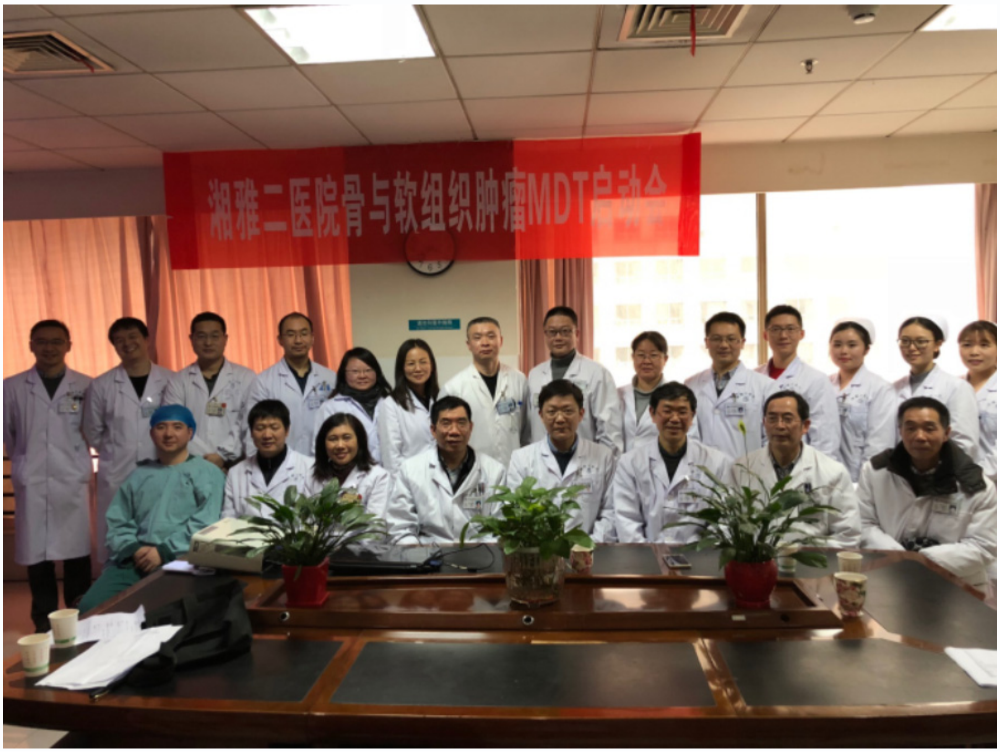

Figure A2. Ongoing MDT Discussion for bone and soft tissue sarcomas

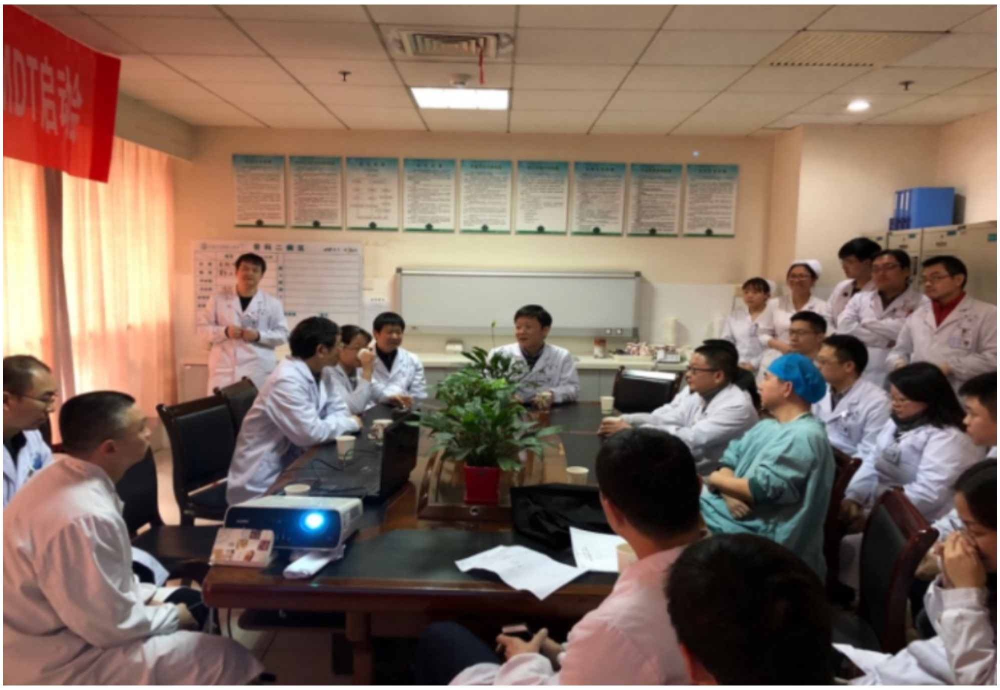

Figure A3.

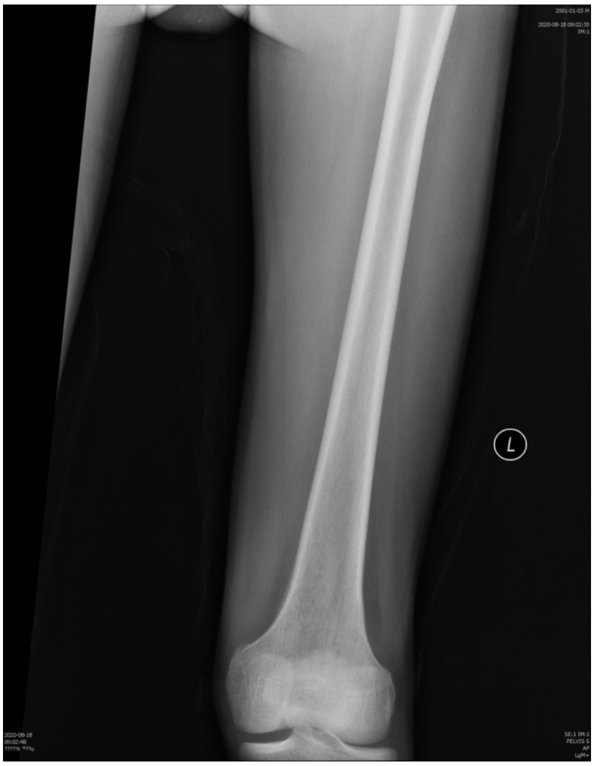

Figure A4.

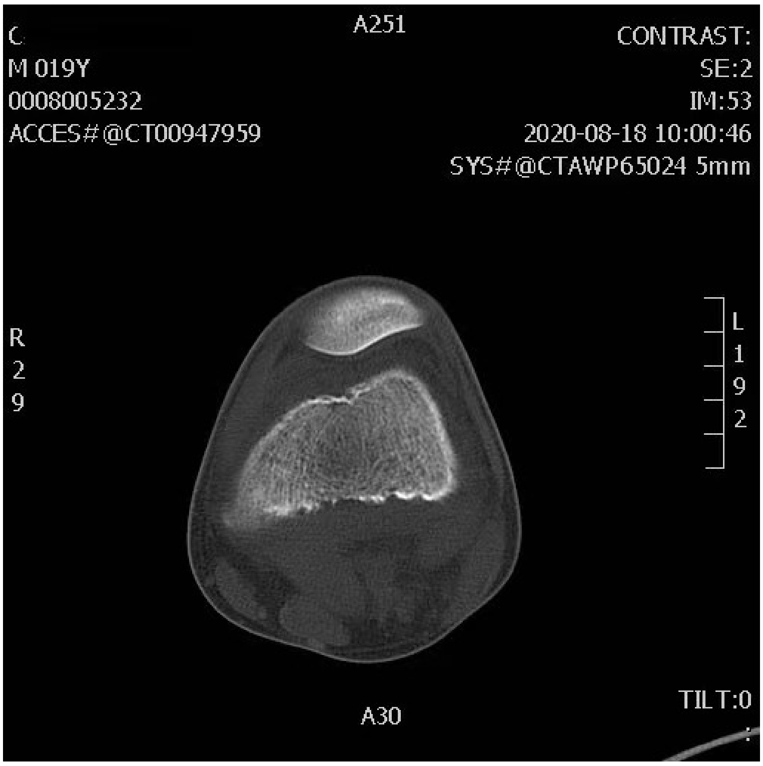

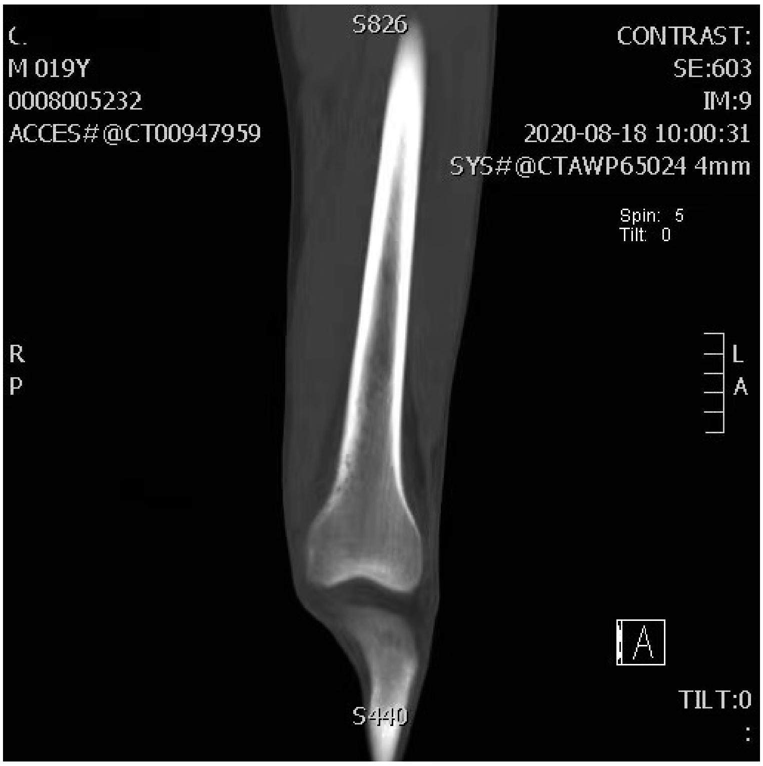
C.
M 019Y
0008005232
ACCES#@CT00947959
S826
CONTRAST:
SE:603
IM:9
2020-08-18 10:00:31
SYS#@CTAWP65024 4mm
Spin: 5
Tilt: 0
R
P
L
A
A
S440
TILT:0

Figure A5.

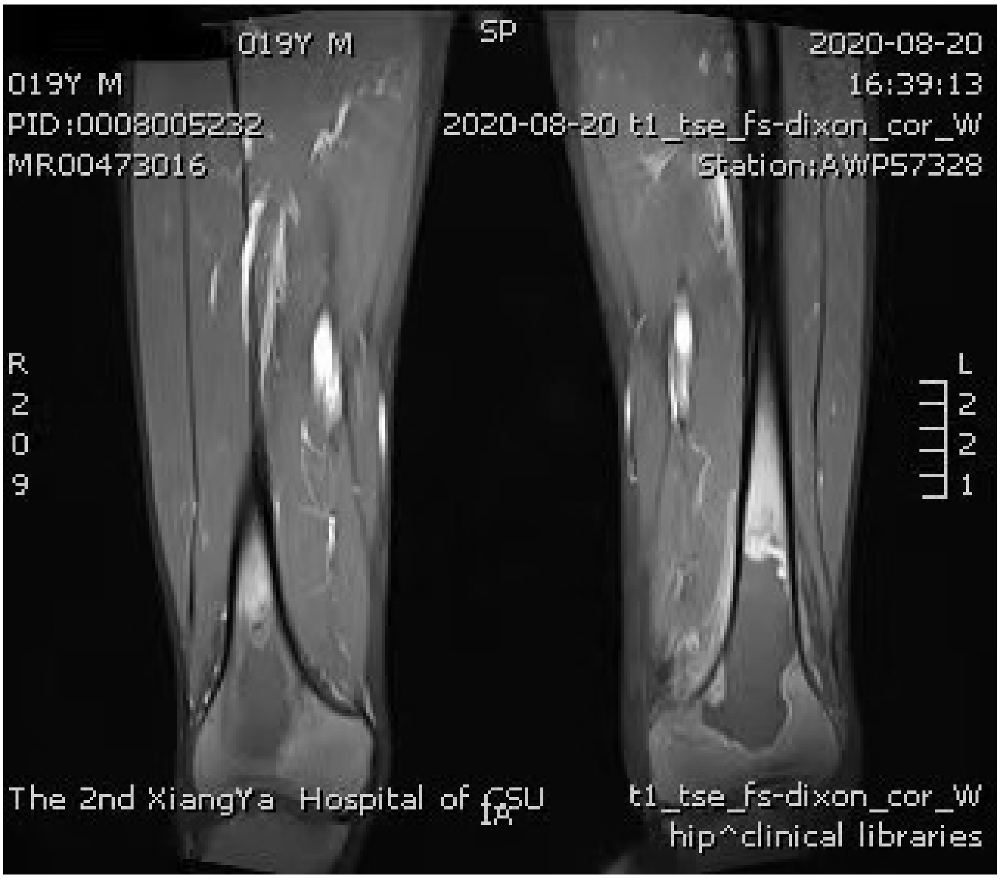
019Y M
SP
2020-08-20
16:39:13
019Y M
PID:0008005232
MR00473016
2020-08-20 t1_tse_fs-dixon_cor_W
Station:AWP57328
R
2
0
9
L
2
2
1
The 2nd XiangYa Hospital of CSU
t1_tse_fs-dixon_cor_W
hip^clinical libraries

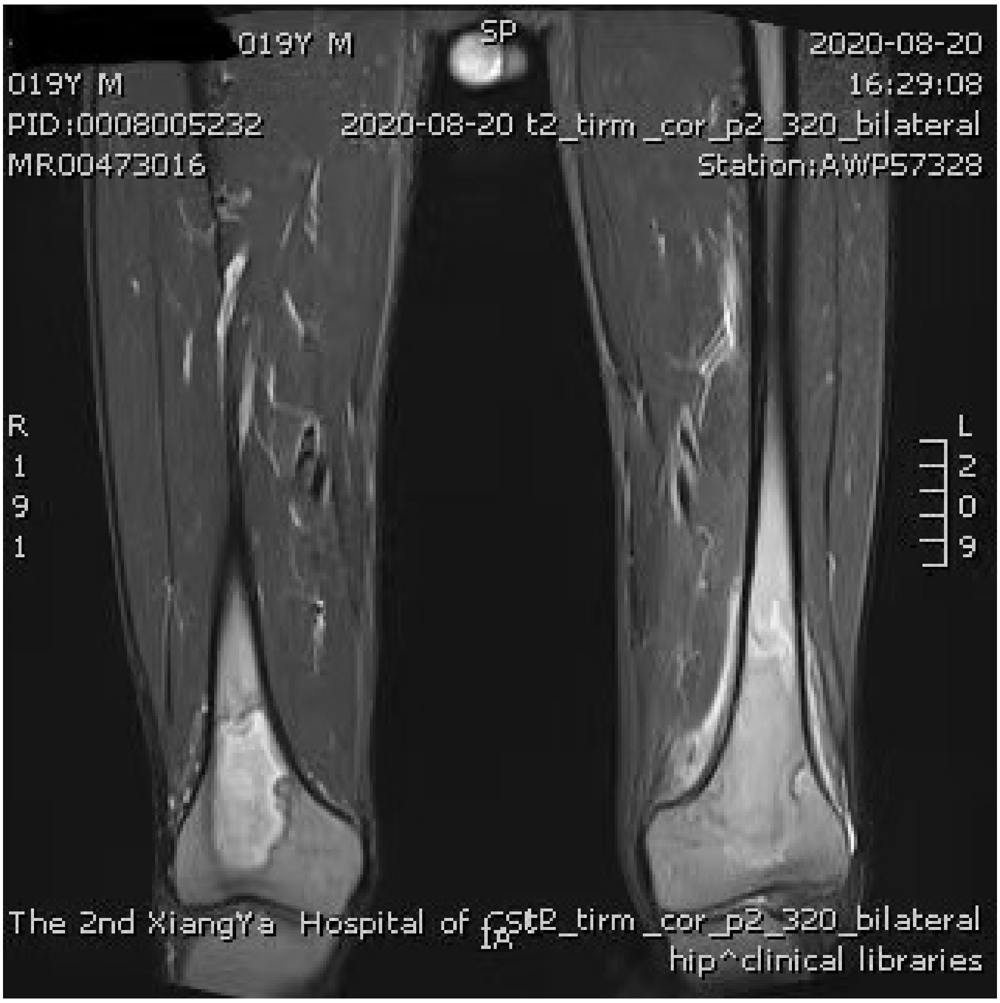
[redacted] 019Y M
SP
2020-08-20
019Y M
16:29:08
PID:0008005232
2020-08-20 t2_tirm _cor_p2_320_bilateral
MR00473016
Station:AWP57328
R
1
9
1
L
2
0
9
The 2nd XiangYa Hospital of CSU
t2_tirm _cor_p2_320_bilateral
IA
hip^clinical libraries
