# Supplementary figures and images for: The Role of a Multidisciplinary Team in the Diagnosis and Treatment of Bone and Soft Tissue Sarcomas: A Single-Center Experience
Source: J Pers Med. 2022 Dec 16;12(12):2079. doi: 10.3390/jpm12122079 (PMC9782466; doi:10.3390/jpm12122079)

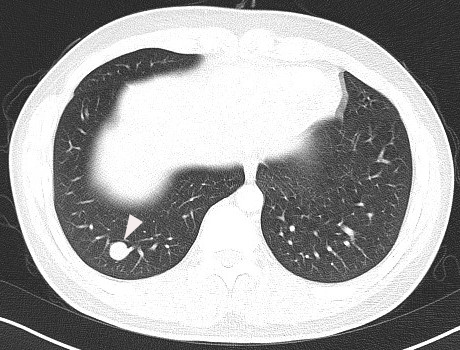

Supplement: Supplementary file 1 [file jpm-12-02079-s001.zip › Supplemental Materials S1/S1a.tif]

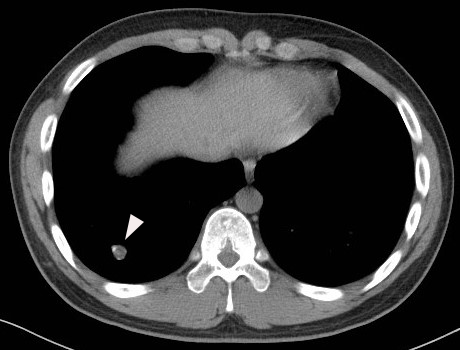

Supplement: Supplementary file 1 [file jpm-12-02079-s001.zip › Supplemental Materials S1/S1b.tif]

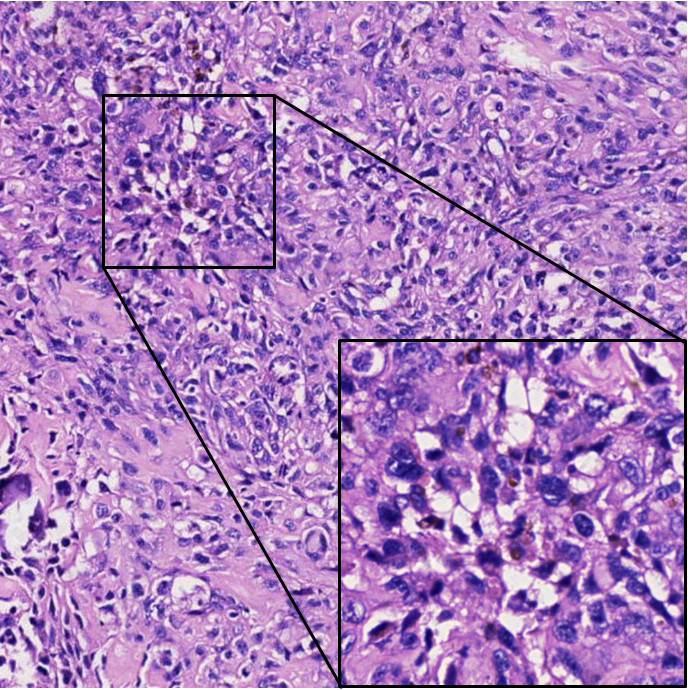

Supplement: Supplementary file 1 [file jpm-12-02079-s001.zip › Supplemental Materials S1/S1c.tif]

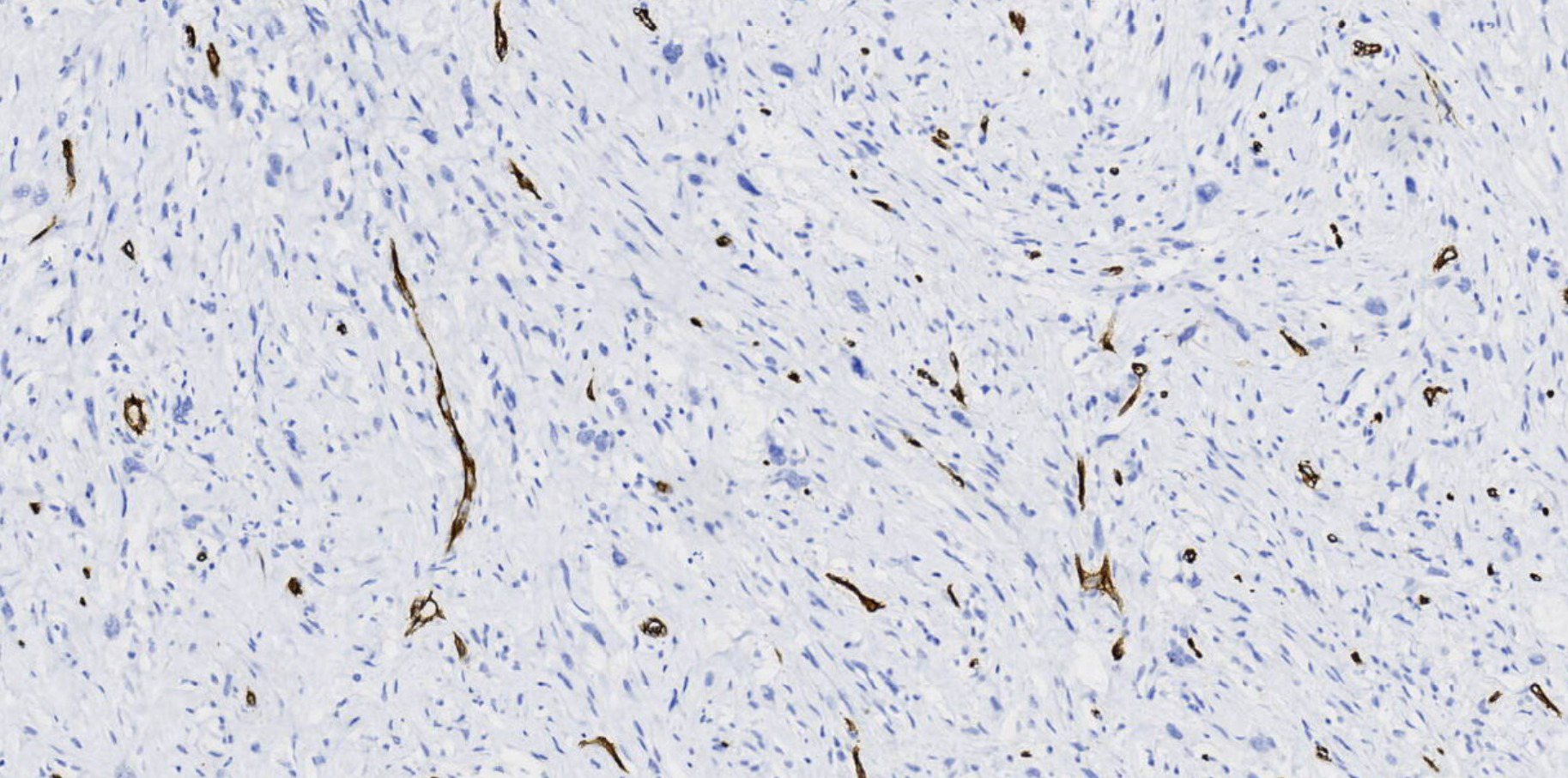

Supplement: Supplementary file 1 [file jpm-12-02079-s001.zip › Supplemental Materials S2/CD34.tif]

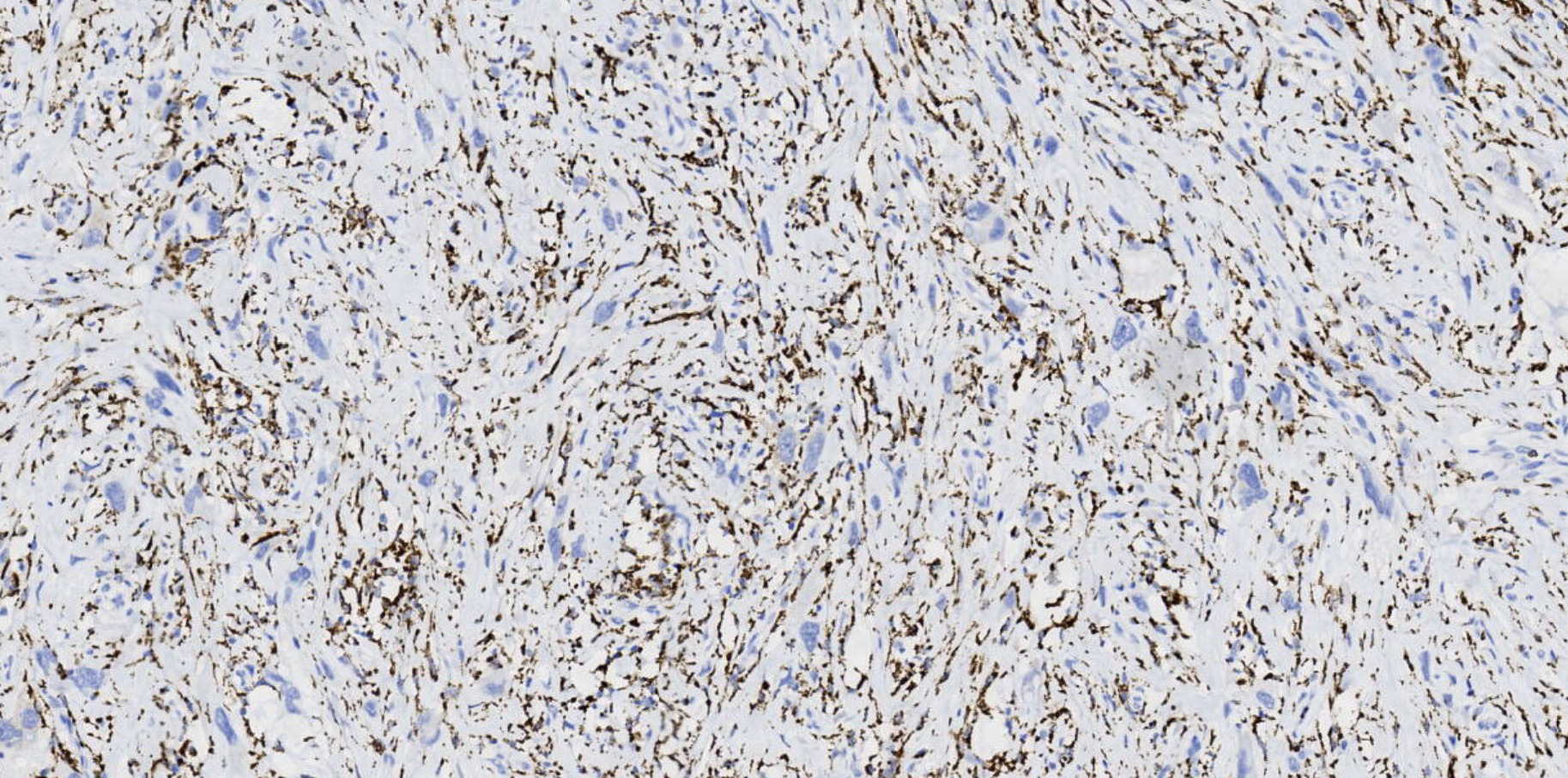

Supplement: Supplementary file 1 [file jpm-12-02079-s001.zip › Supplemental Materials S2/CD68.tif]

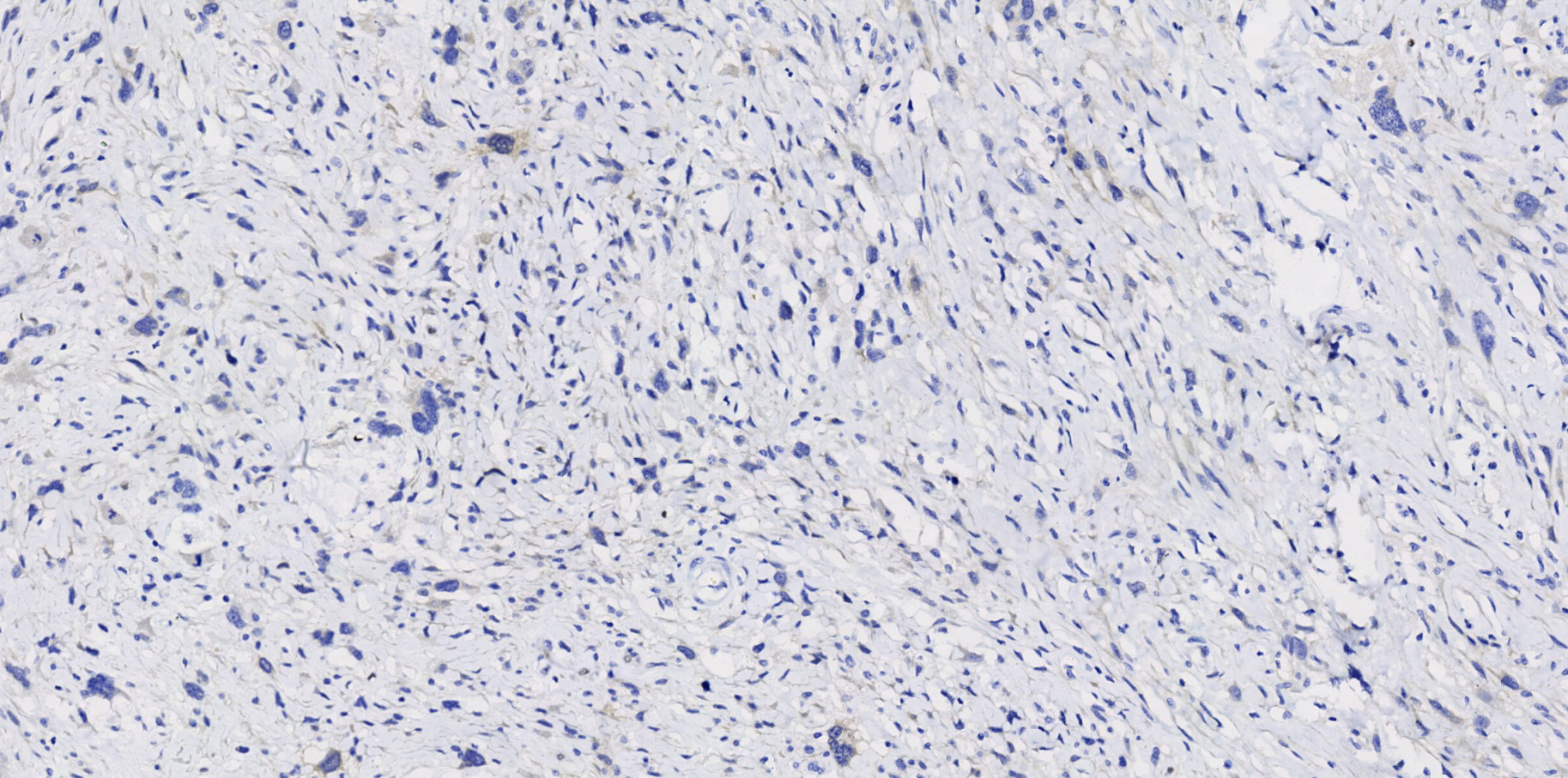

Supplement: Supplementary file 1 [file jpm-12-02079-s001.zip › Supplemental Materials S2/CDK4.tif]

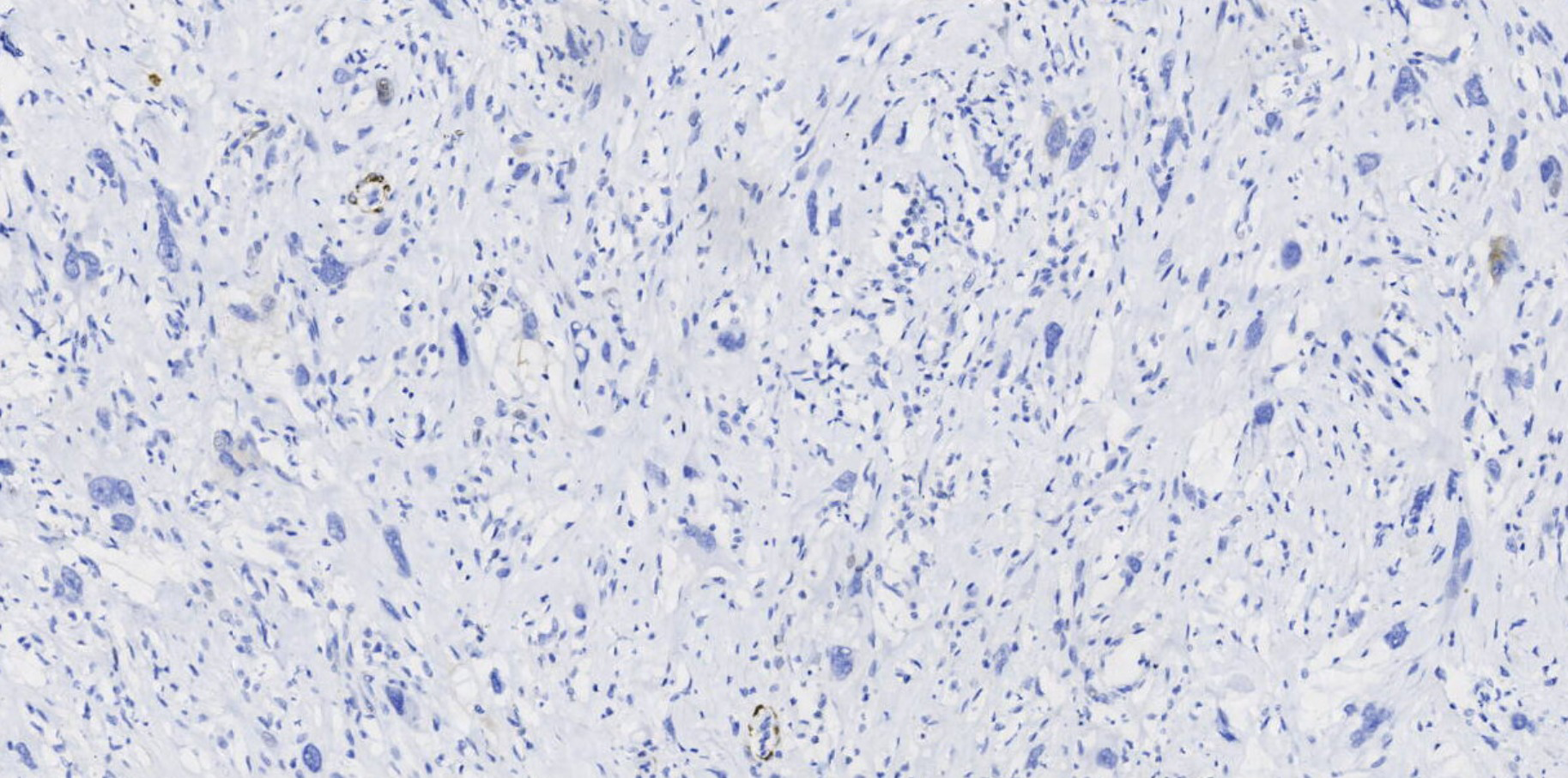

Supplement: Supplementary file 1 [file jpm-12-02079-s001.zip › Supplemental Materials S2/DES b.tif]

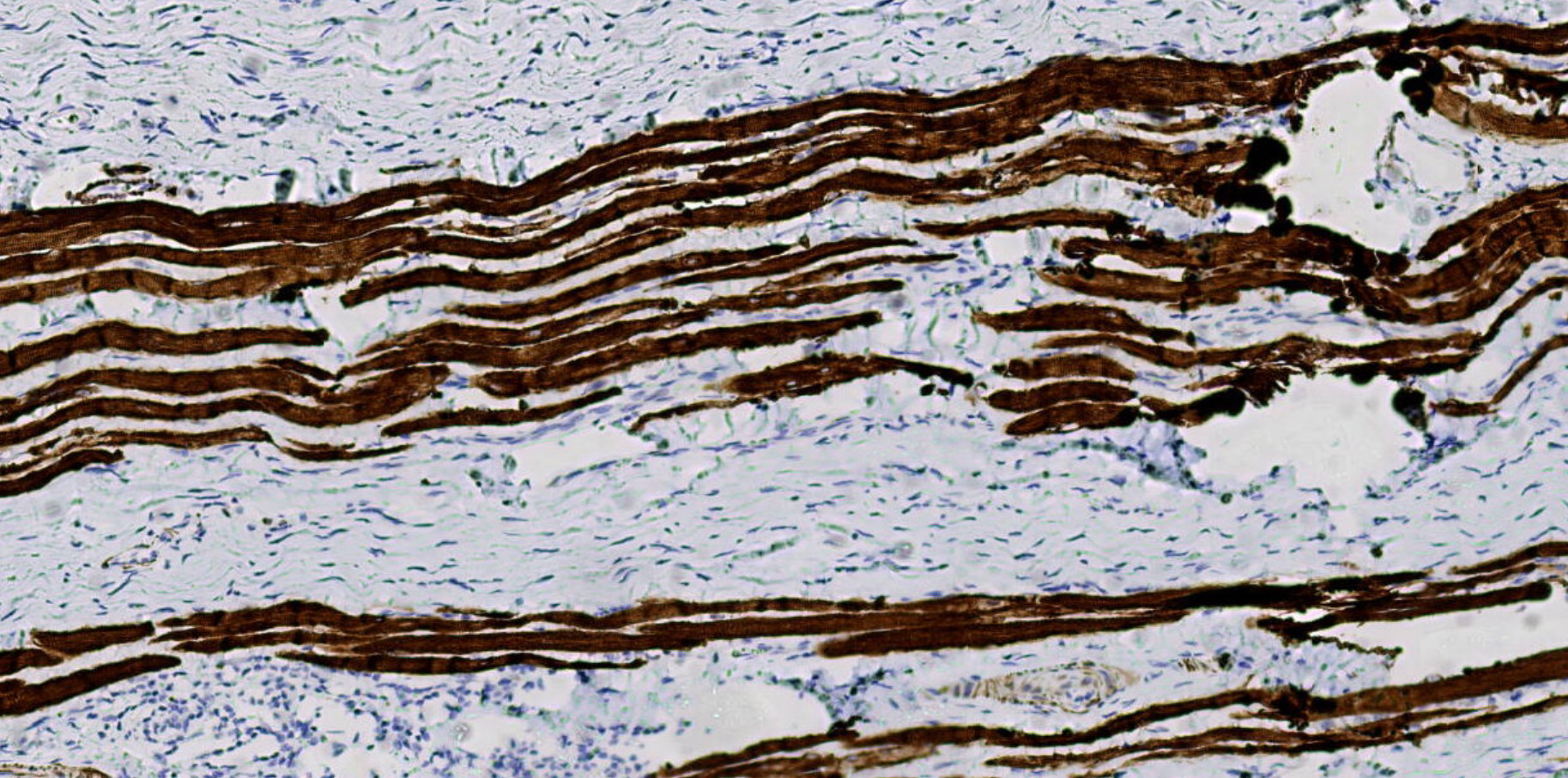

Supplement: Supplementary file 1 [file jpm-12-02079-s001.zip › Supplemental Materials S2/DES.tif]

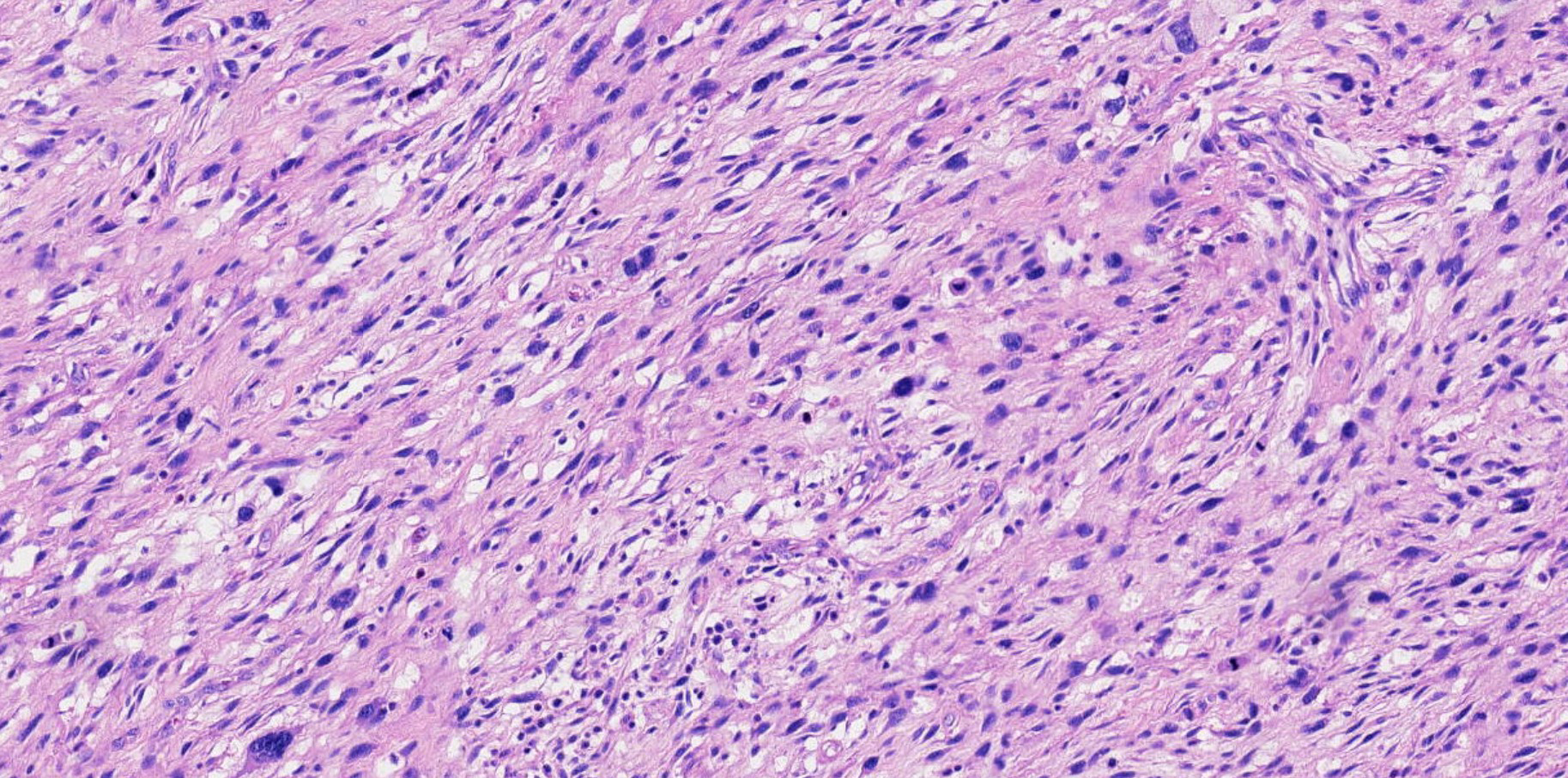

Supplement: Supplementary file 1 [file jpm-12-02079-s001.zip › Supplemental Materials S2/HE 100+.tif]

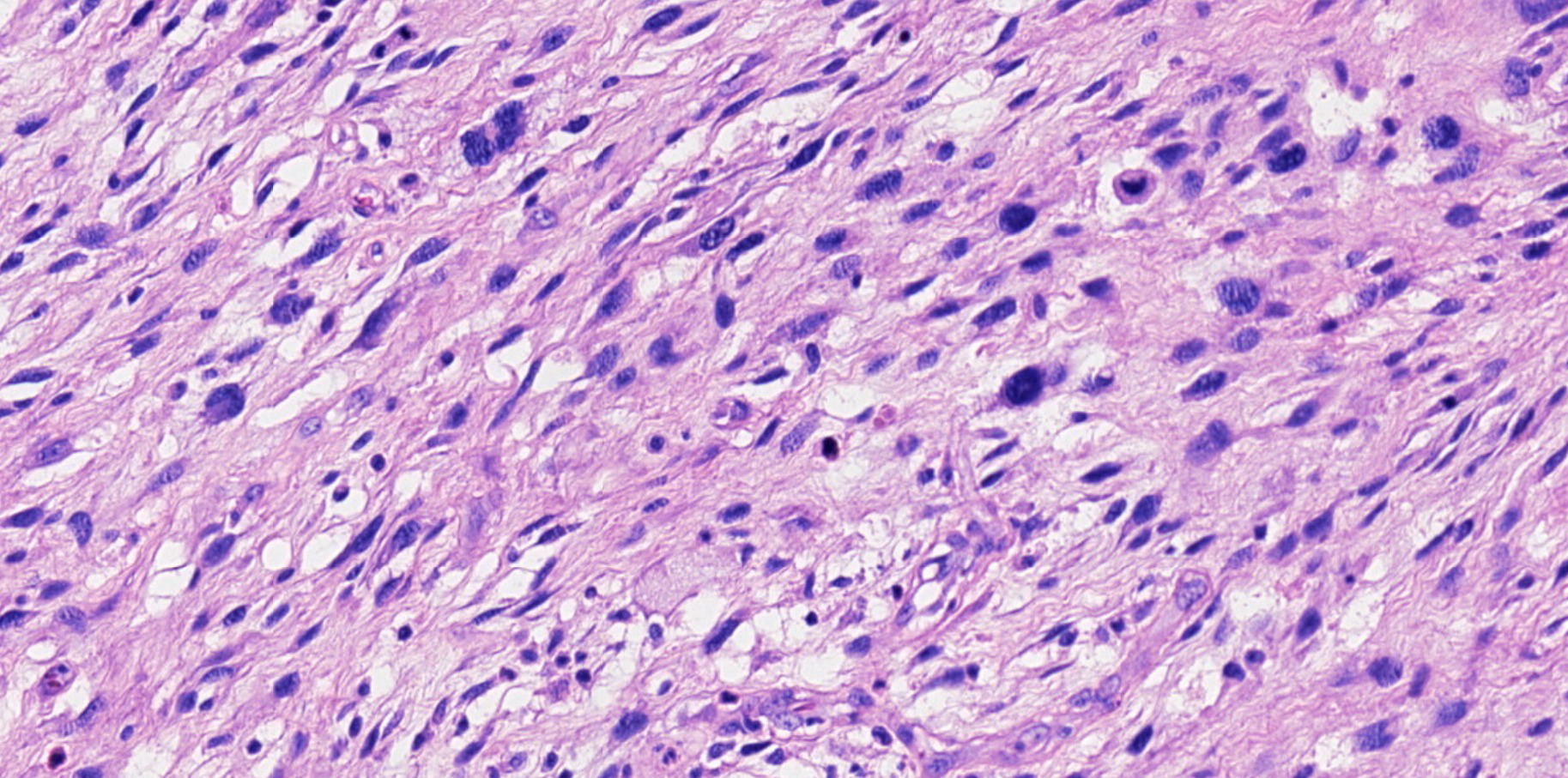

Supplement: Supplementary file 1 [file jpm-12-02079-s001.zip › Supplemental Materials S2/HE 200+.tif]

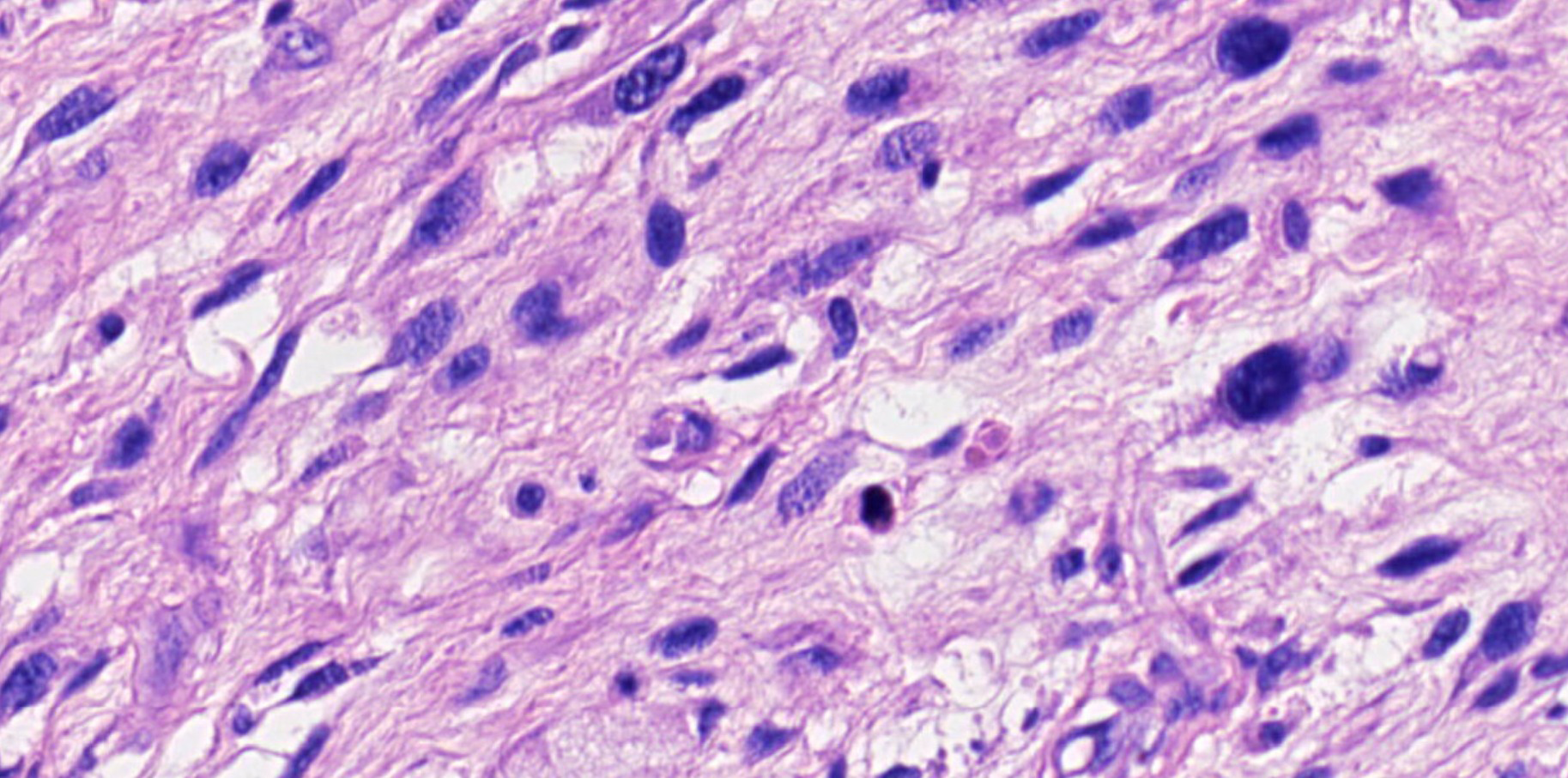

Supplement: Supplementary file 1 [file jpm-12-02079-s001.zip › Supplemental Materials S2/HE 400+.tif]

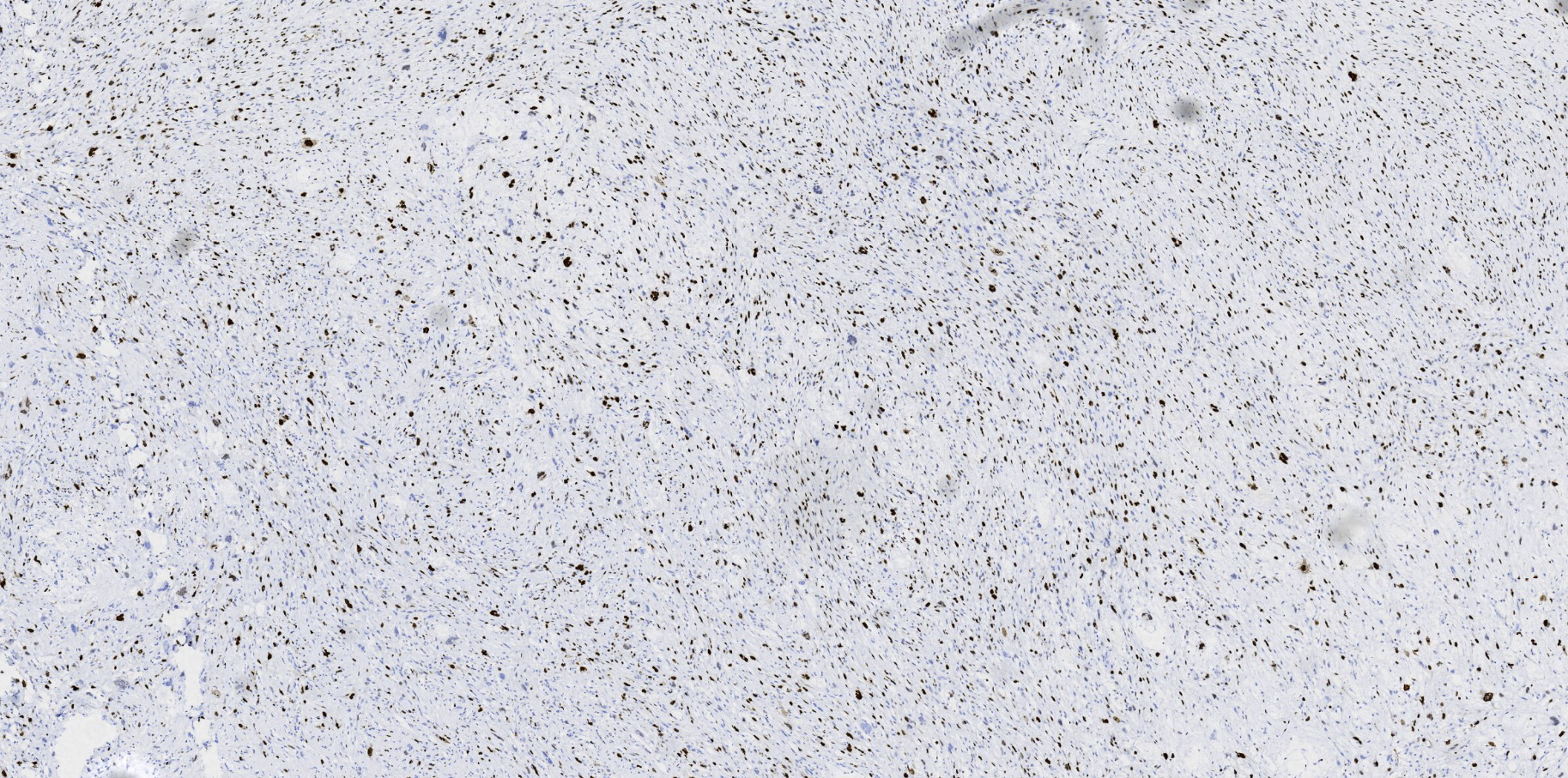

Supplement: Supplementary file 1 [file jpm-12-02079-s001.zip › Supplemental Materials S2/Ki67.tif]

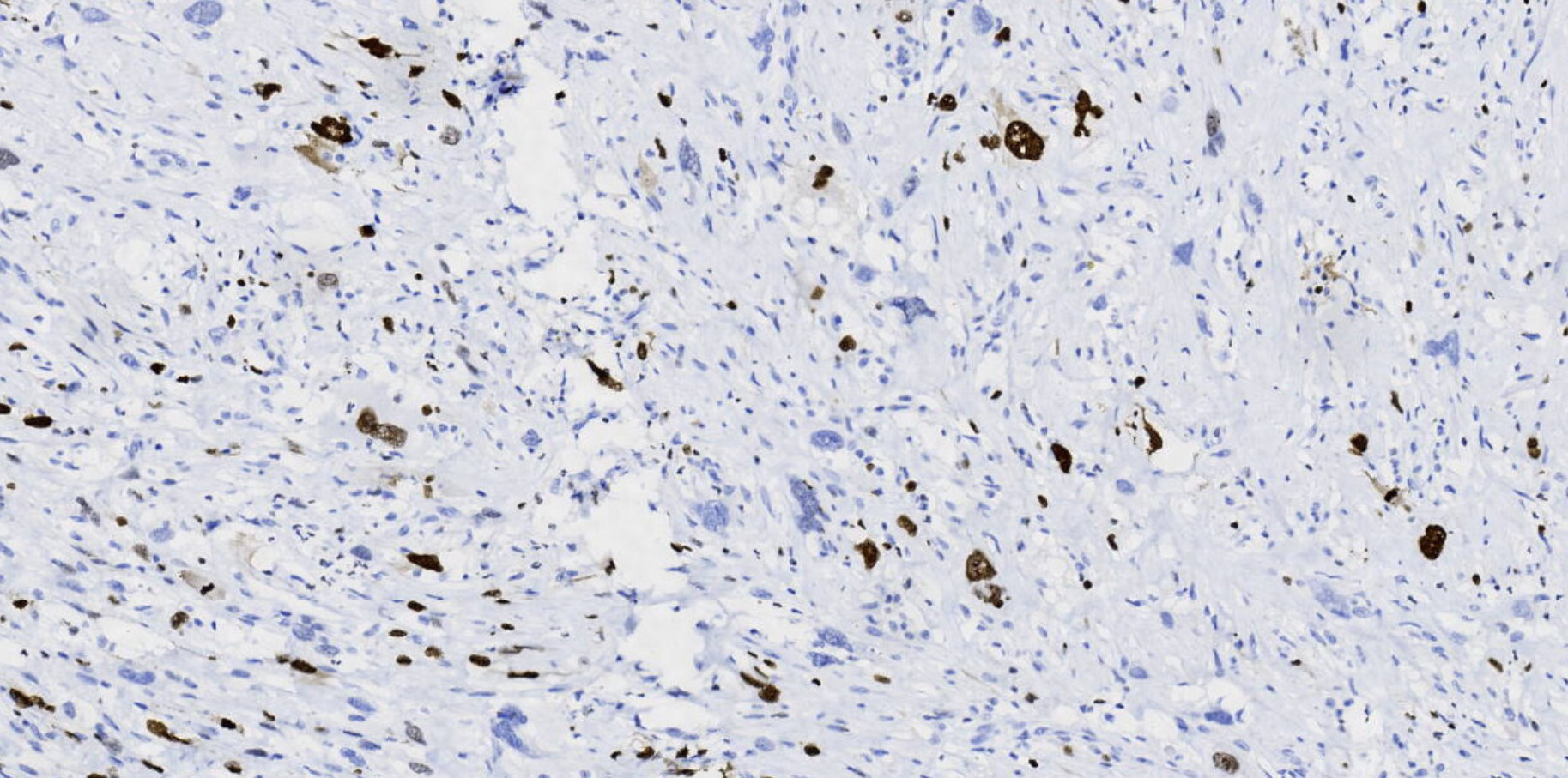

Supplement: Supplementary file 1 [file jpm-12-02079-s001.zip › Supplemental Materials S2/Ki-67.tif]

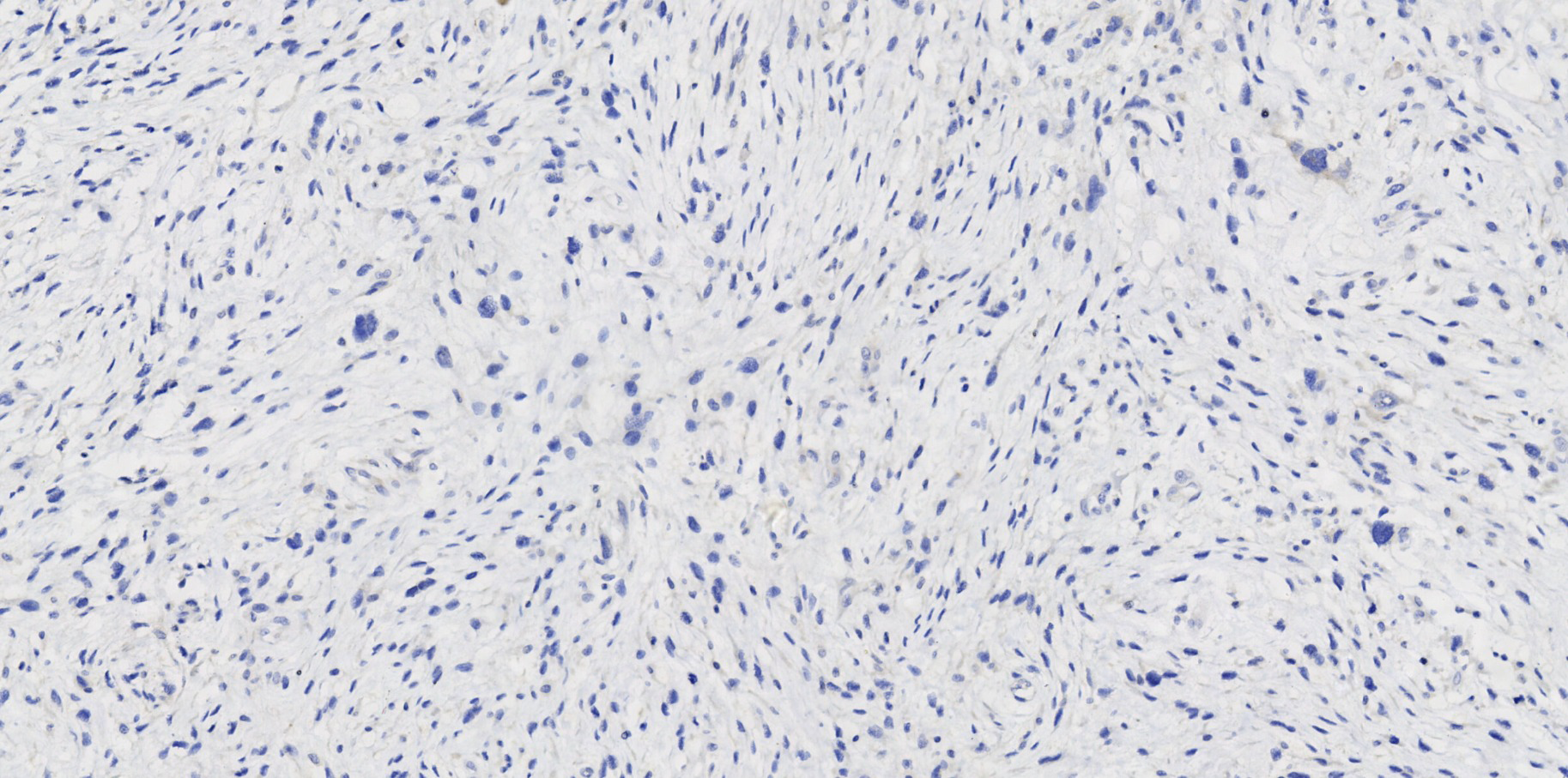

Supplement: Supplementary file 1 [file jpm-12-02079-s001.zip › Supplemental Materials S2/MDM2.tif]

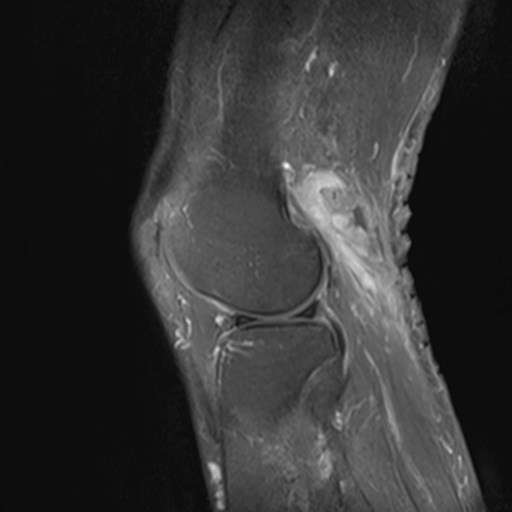

Supplement: Supplementary file 1 [file jpm-12-02079-s001.zip › Supplemental Materials S2/MRI 1.tif]

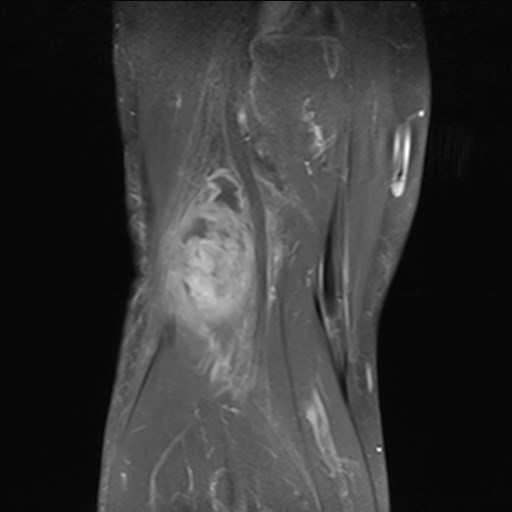

Supplement: Supplementary file 1 [file jpm-12-02079-s001.zip › Supplemental Materials S2/MRI 2.tif]

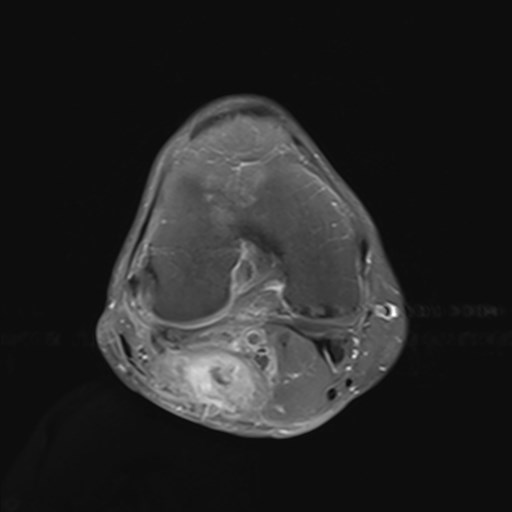

Supplement: Supplementary file 1 [file jpm-12-02079-s001.zip › Supplemental Materials S2/MRI 3.tif]

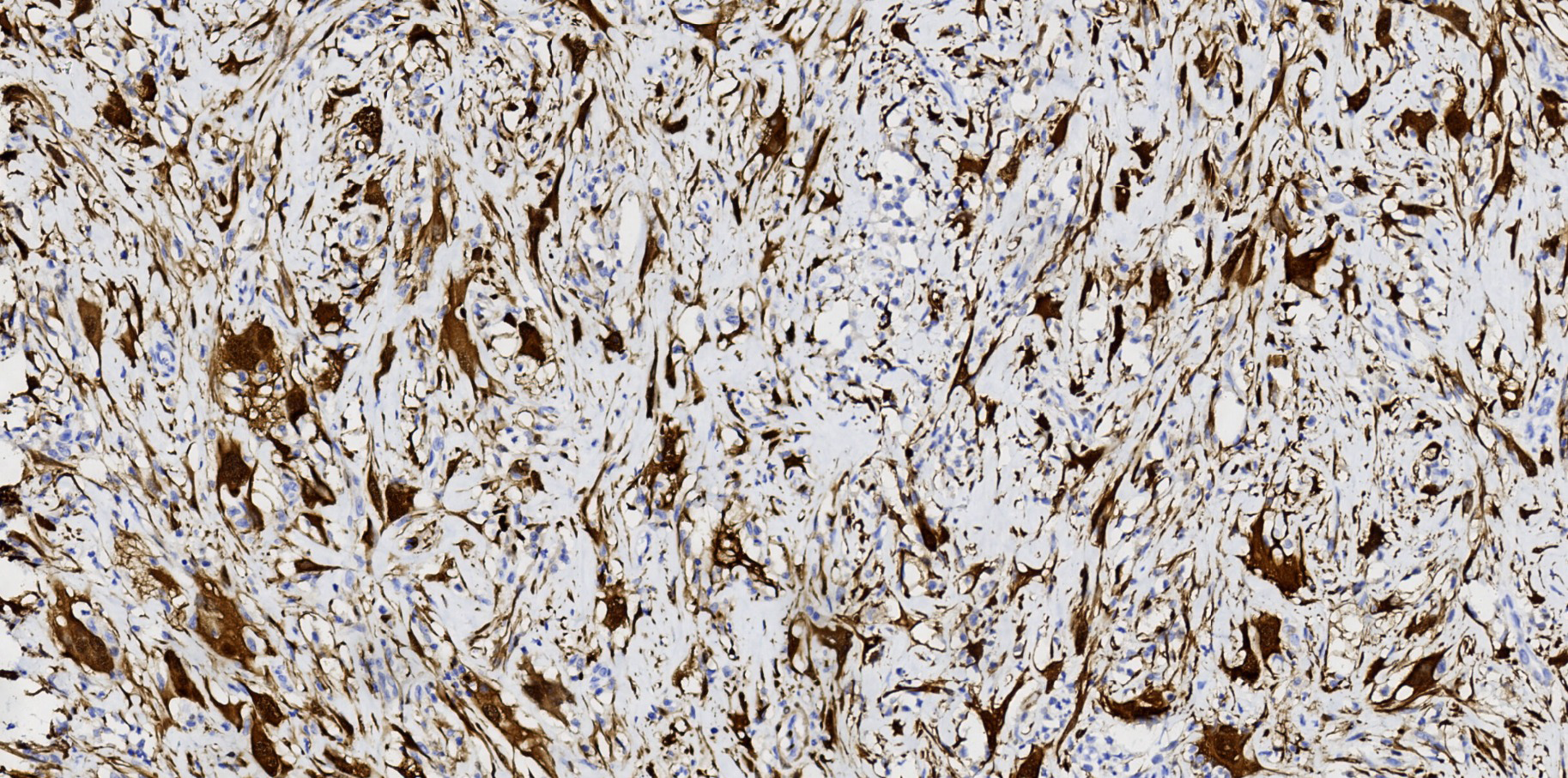

Supplement: Supplementary file 1 [file jpm-12-02079-s001.zip › Supplemental Materials S2/P16.tif]

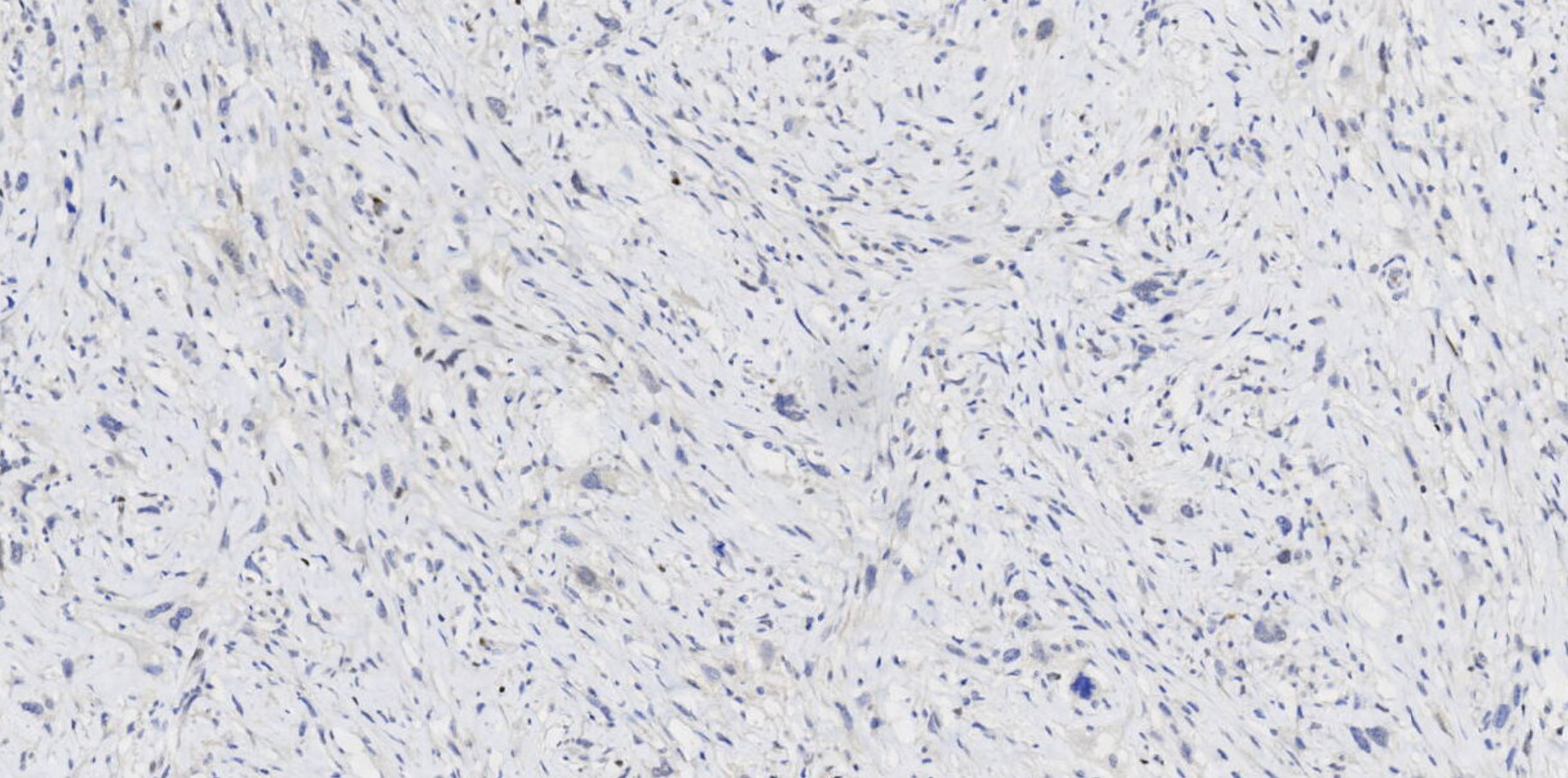

Supplement: Supplementary file 1 [file jpm-12-02079-s001.zip › Supplemental Materials S2/P53.tif]

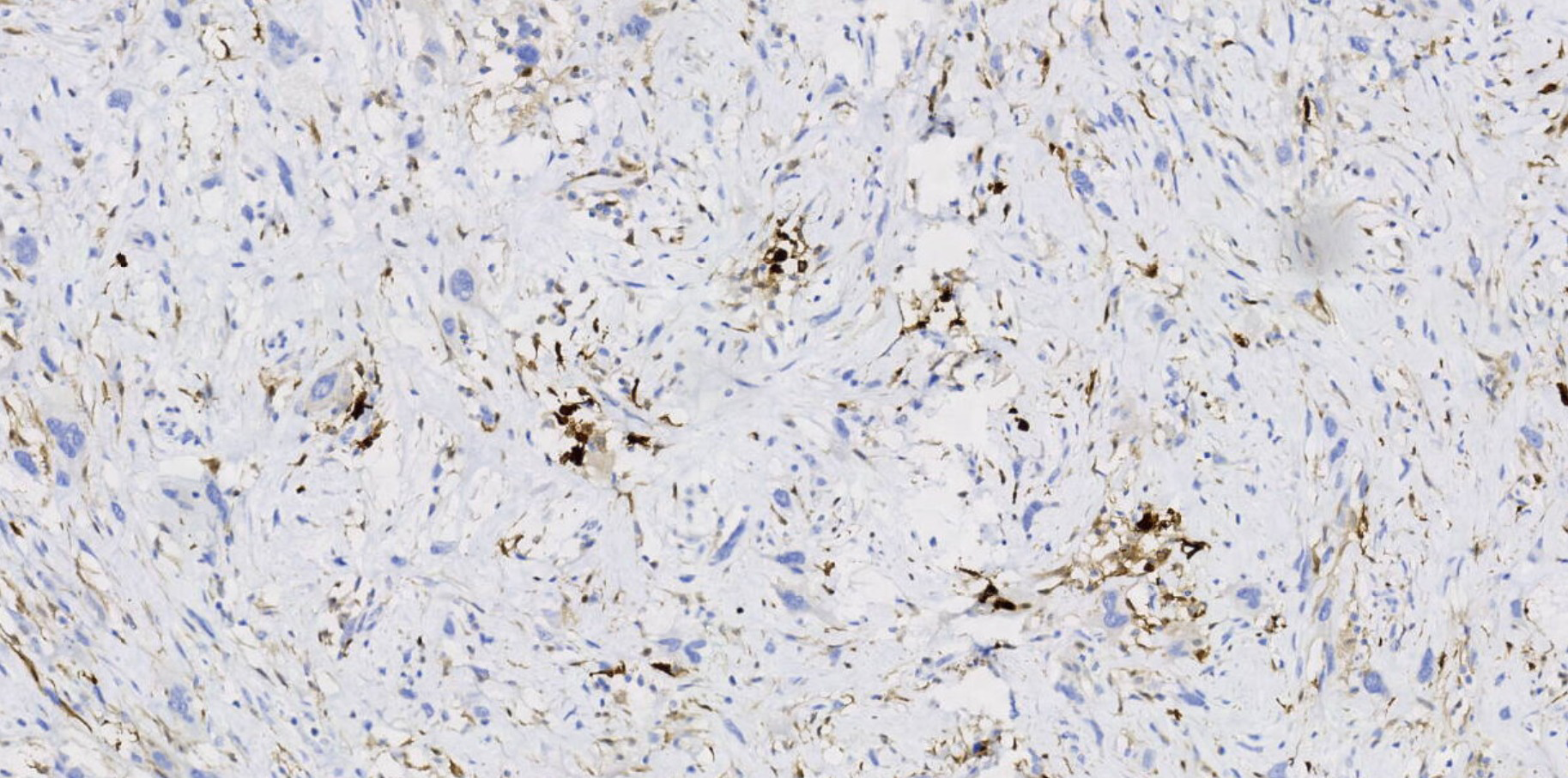

Supplement: Supplementary file 1 [file jpm-12-02079-s001.zip › Supplemental Materials S2/S100.tif]

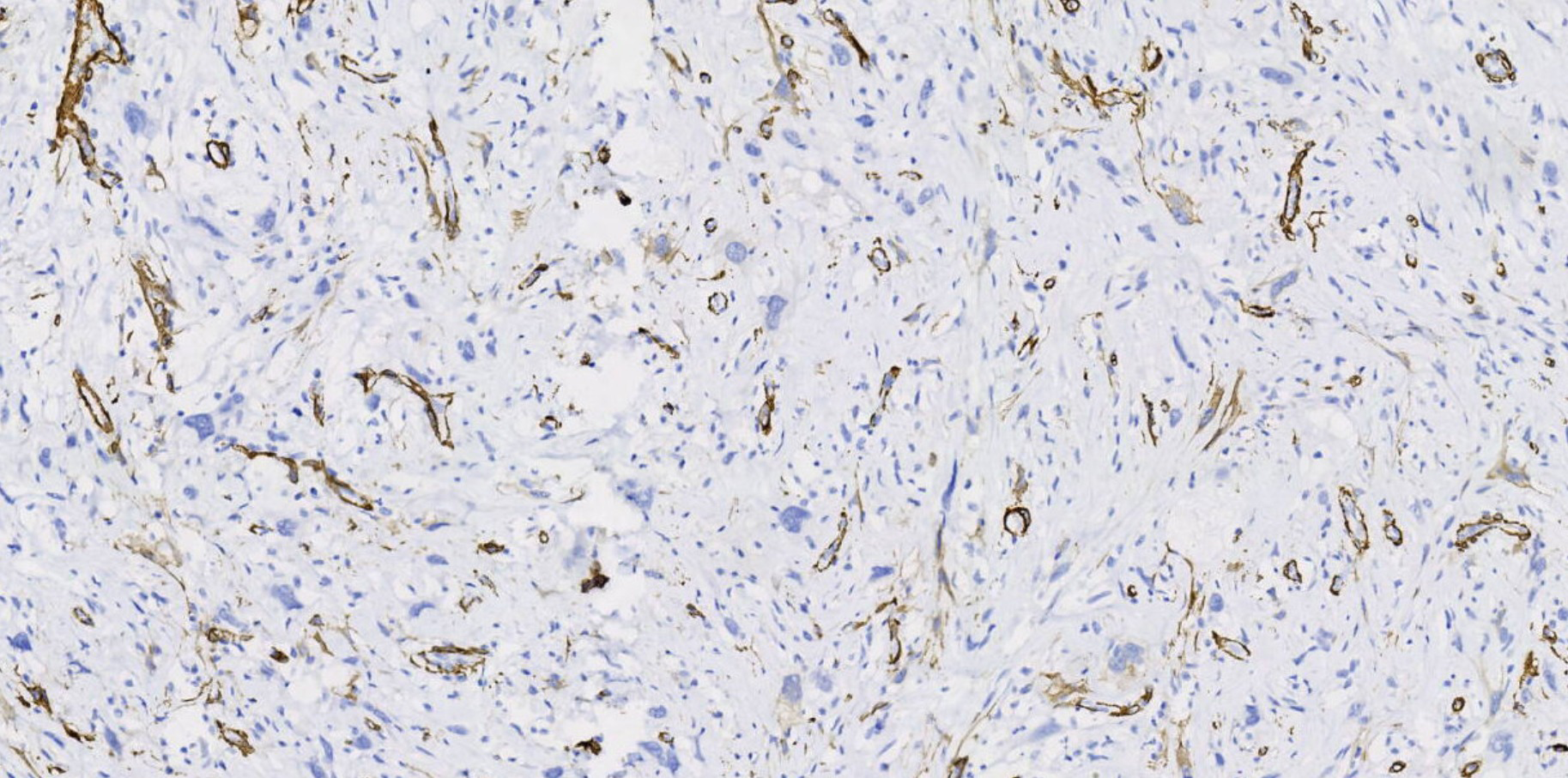

Supplement: Supplementary file 1 [file jpm-12-02079-s001.zip › Supplemental Materials S2/SMA.tif]

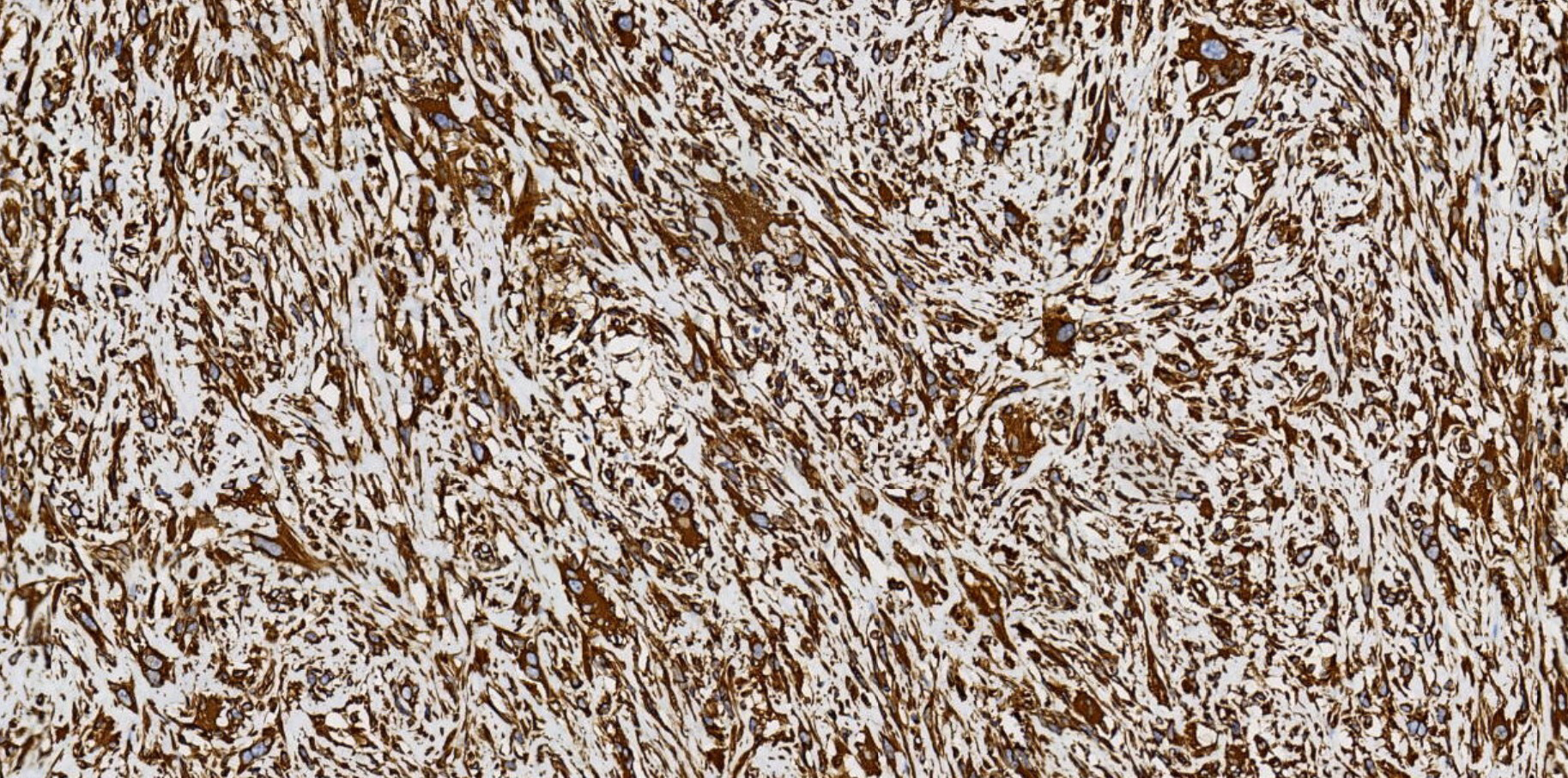

Supplement: Supplementary file 1 [file jpm-12-02079-s001.zip › Supplemental Materials S2/Vim.tif]

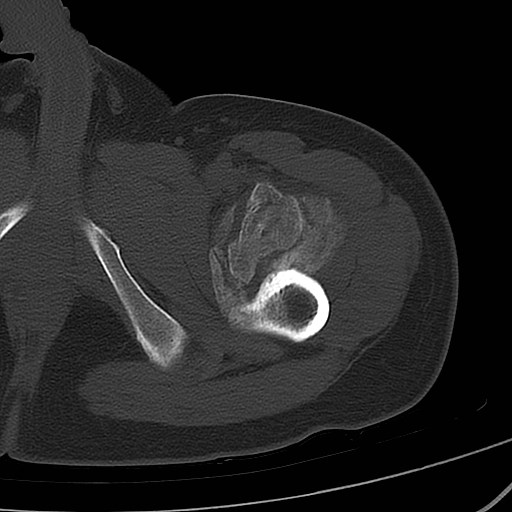

Supplement: Supplementary file 1 [file jpm-12-02079-s001.zip › Supplemental Materials S3/S3.tif]

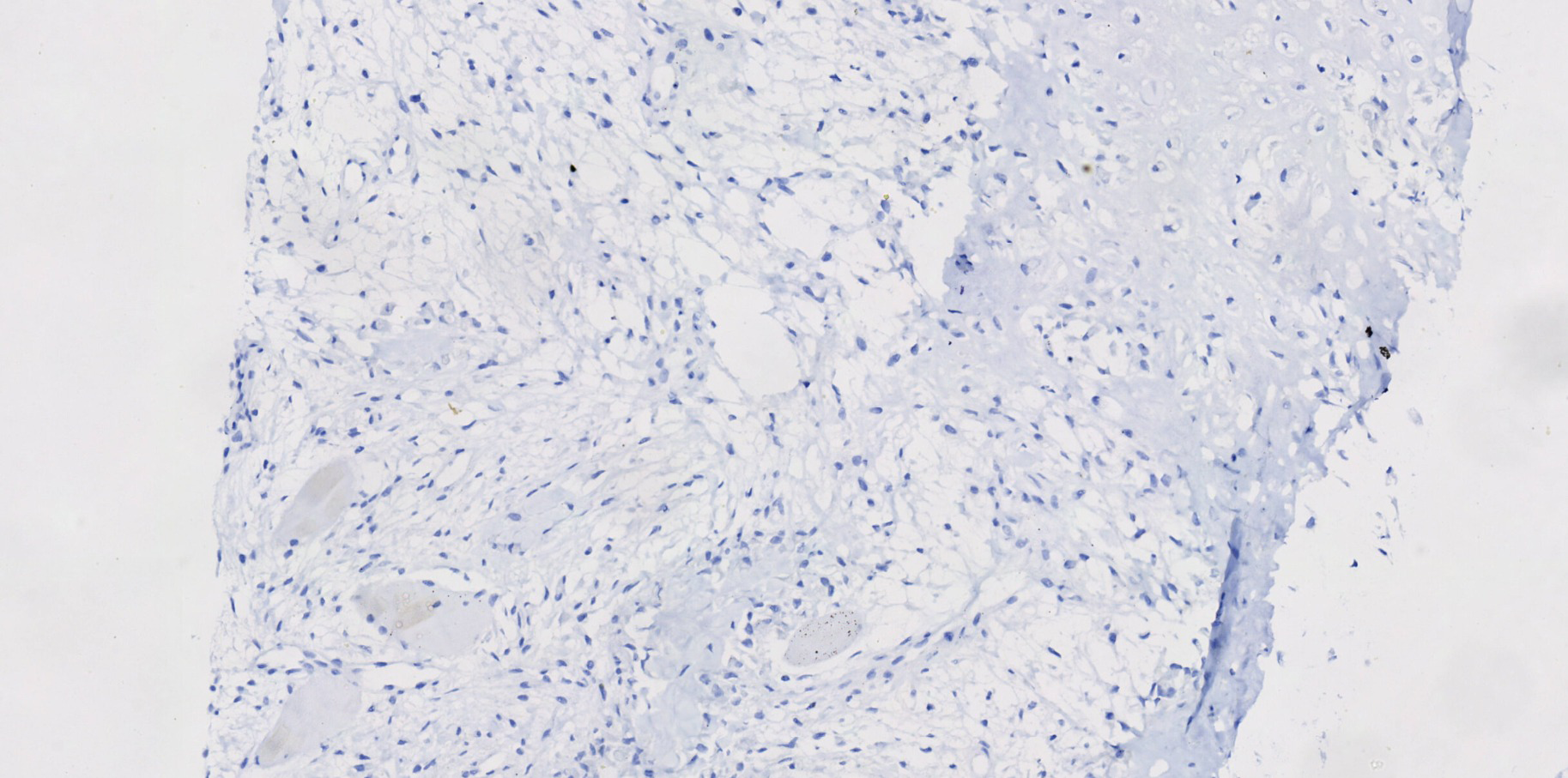

Supplement: Supplementary file 1 [file jpm-12-02079-s001.zip › Supplemental Materials S3/S3-ALK p80.tif]

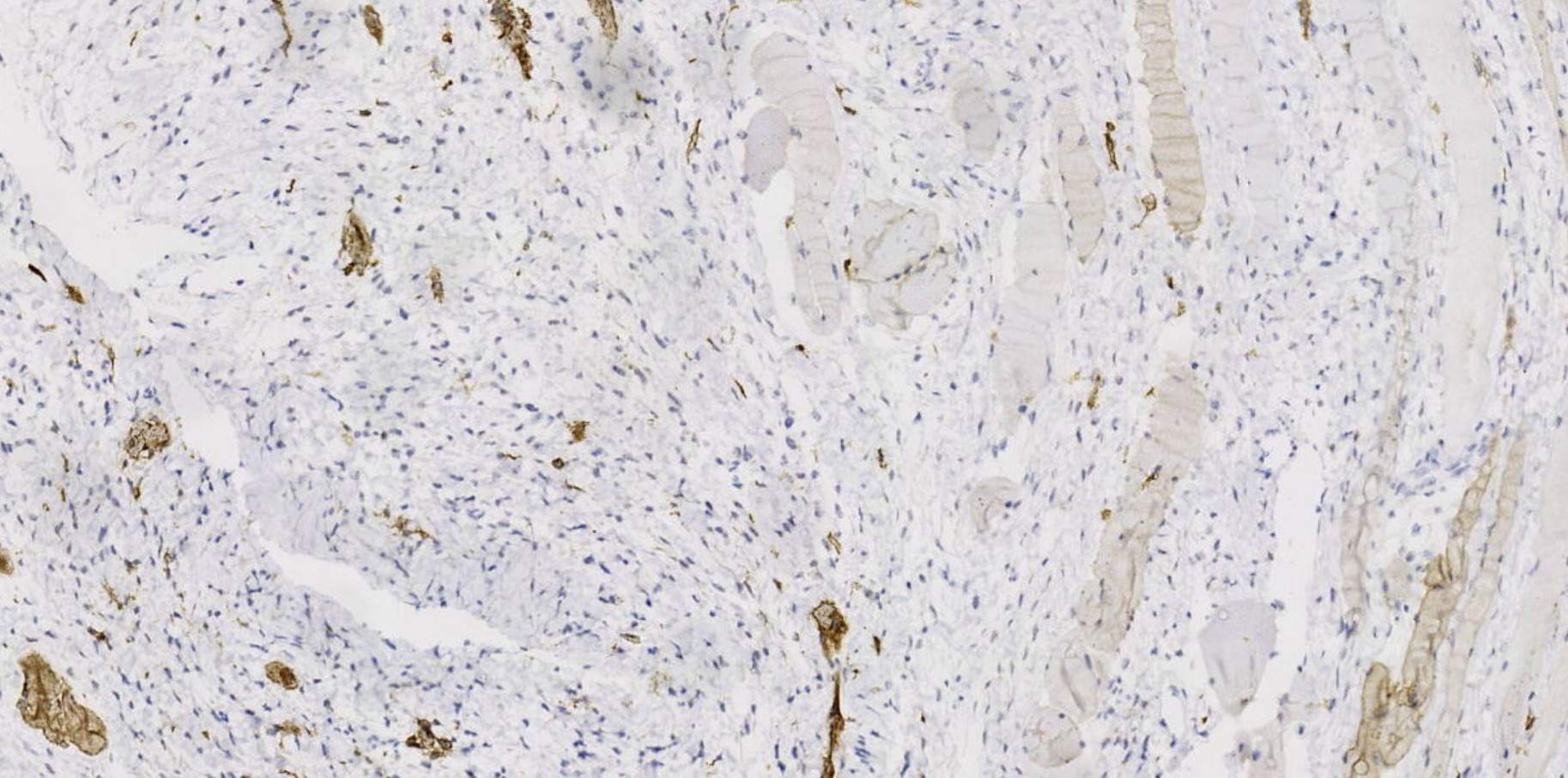

Supplement: Supplementary file 1 [file jpm-12-02079-s001.zip › Supplemental Materials S3/S3-CD56.tif]

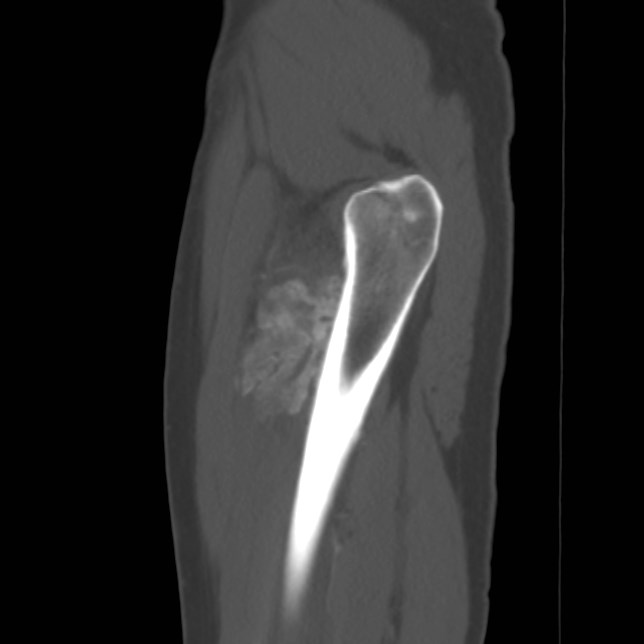

Supplement: Supplementary file 1 [file jpm-12-02079-s001.zip › Supplemental Materials S3/S3-CT1.tif]

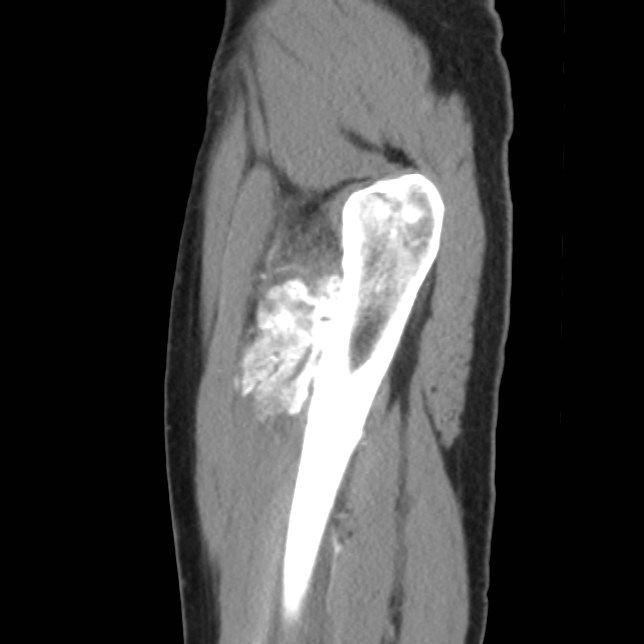

Supplement: Supplementary file 1 [file jpm-12-02079-s001.zip › Supplemental Materials S3/S3-CT2.tif]

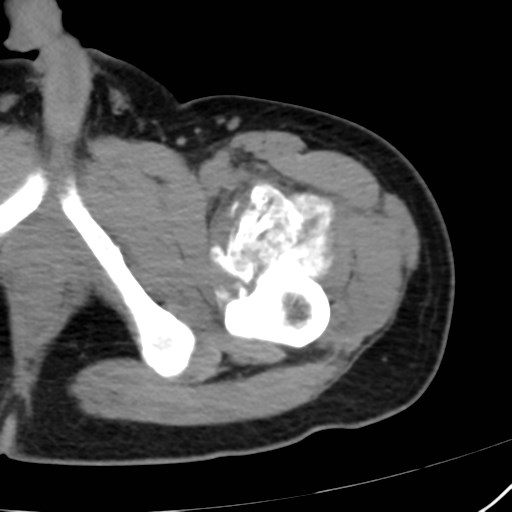

Supplement: Supplementary file 1 [file jpm-12-02079-s001.zip › Supplemental Materials S3/S3-CT4.tif]

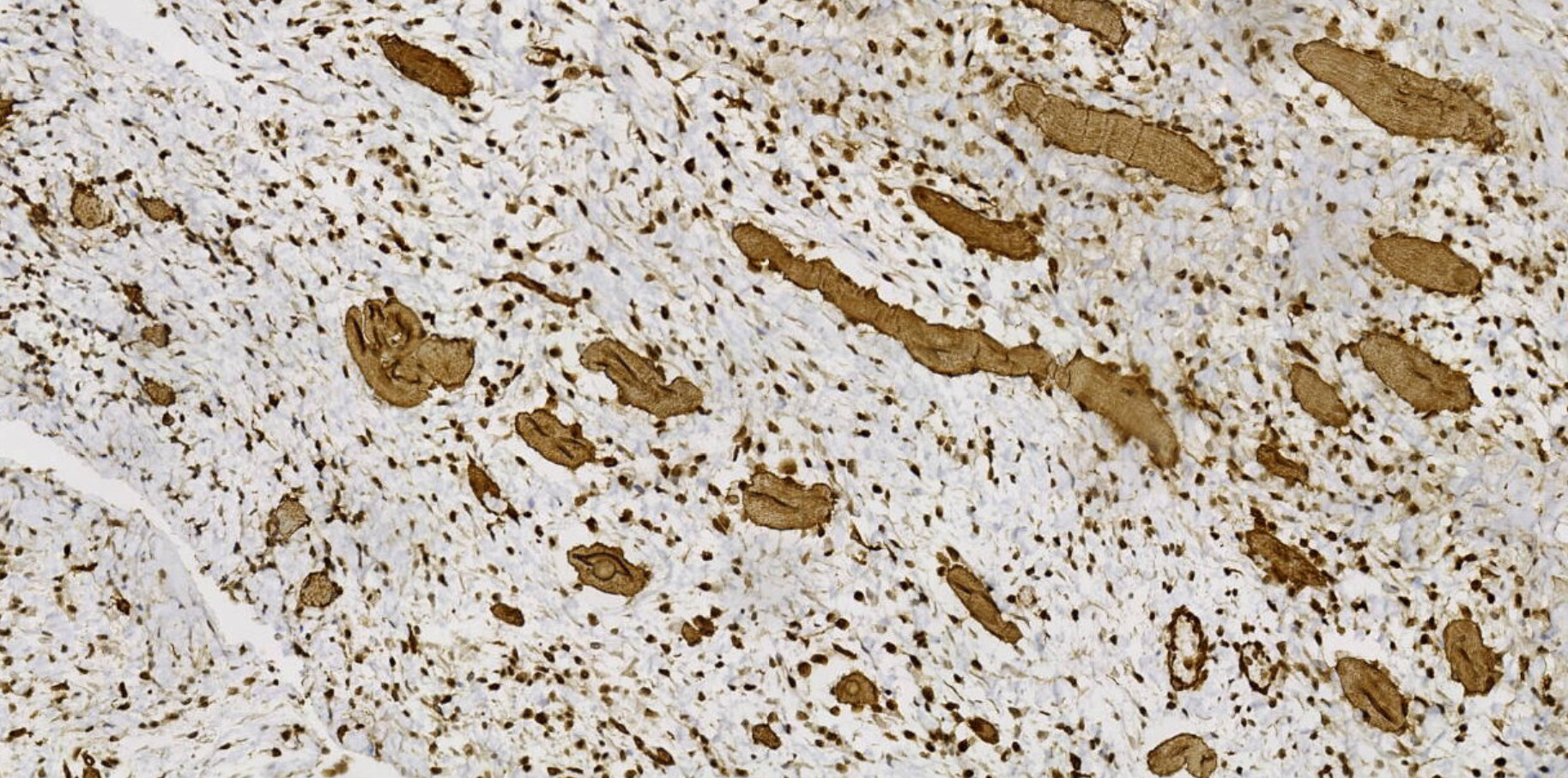

Supplement: Supplementary file 1 [file jpm-12-02079-s001.zip › Supplemental Materials S3/S3-Desmin.tif]

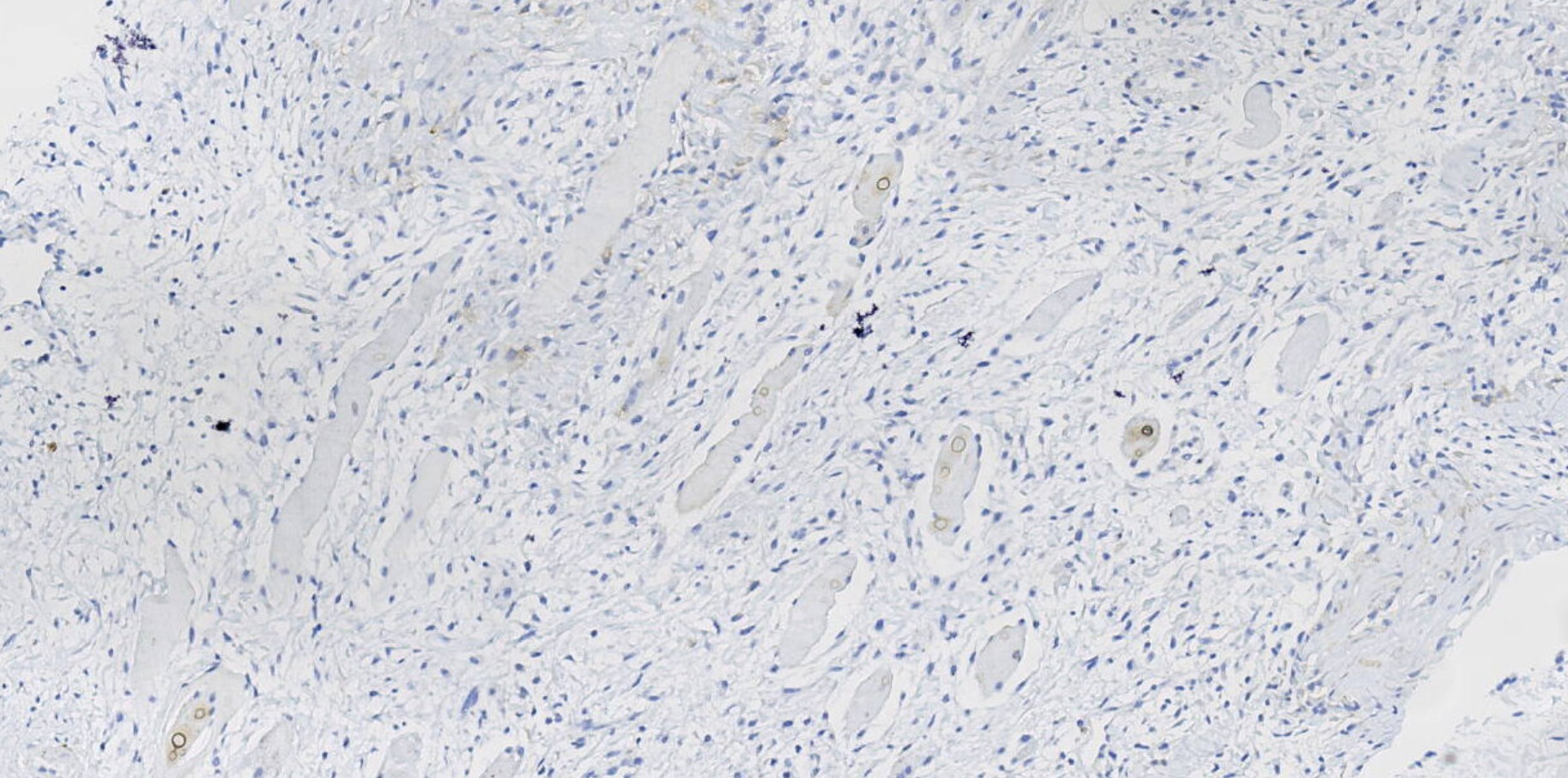

Supplement: Supplementary file 1 [file jpm-12-02079-s001.zip › Supplemental Materials S3/S3-EMA.tif]

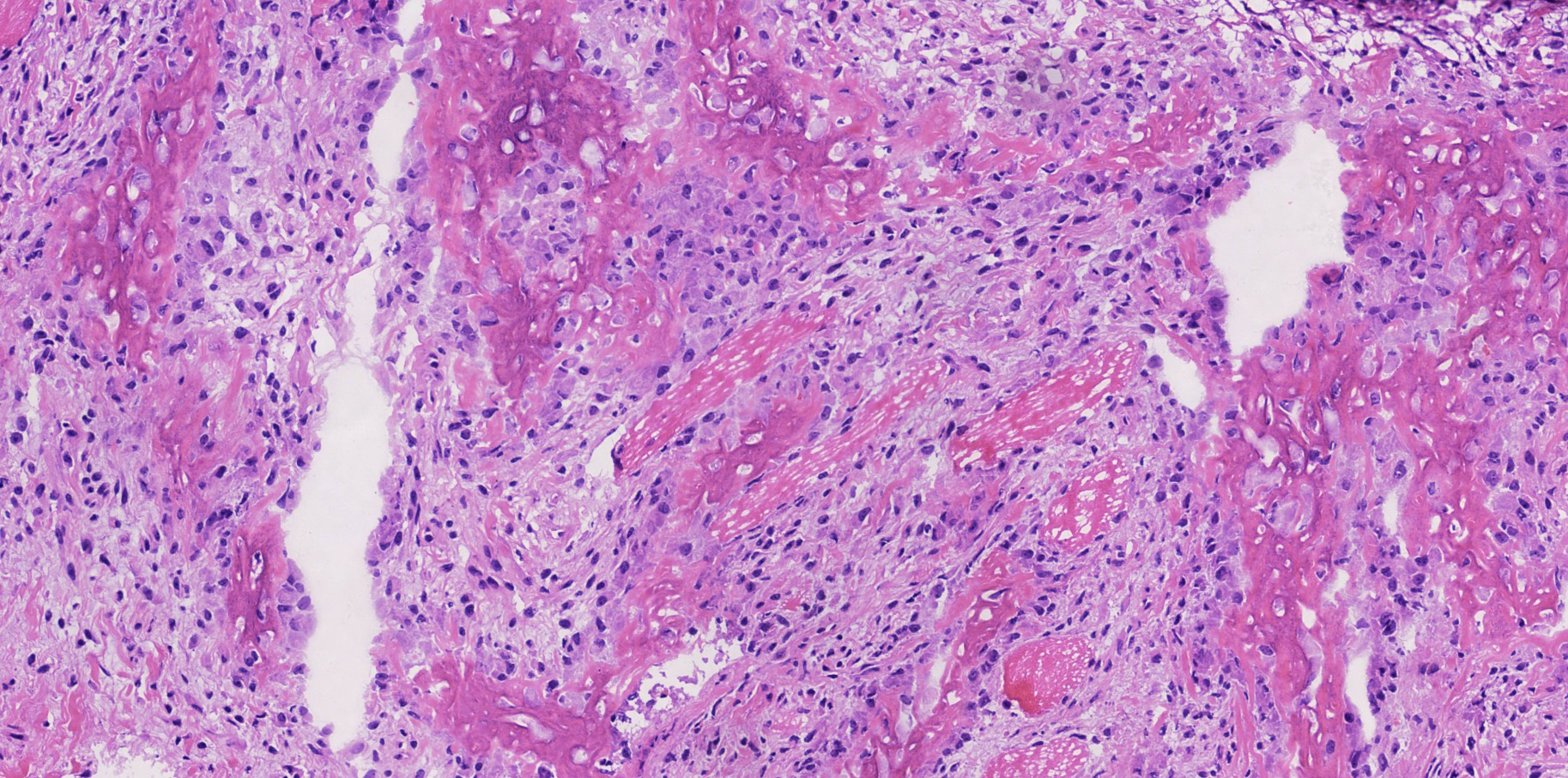

Supplement: Supplementary file 1 [file jpm-12-02079-s001.zip › Supplemental Materials S3/S3-Frozen section HE100+.tif]

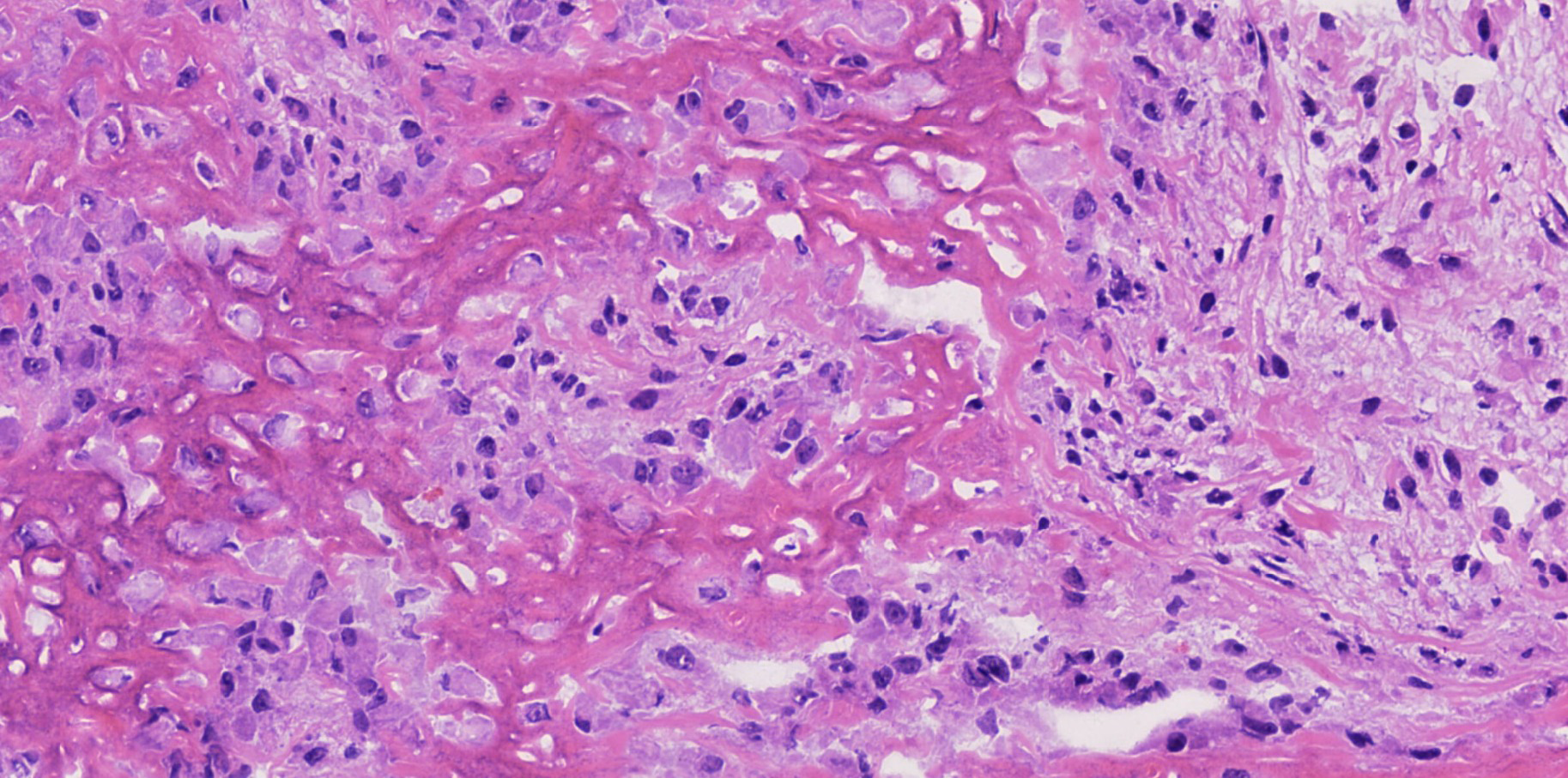

Supplement: Supplementary file 1 [file jpm-12-02079-s001.zip › Supplemental Materials S3/S3-Frozen section HE200+.tif]

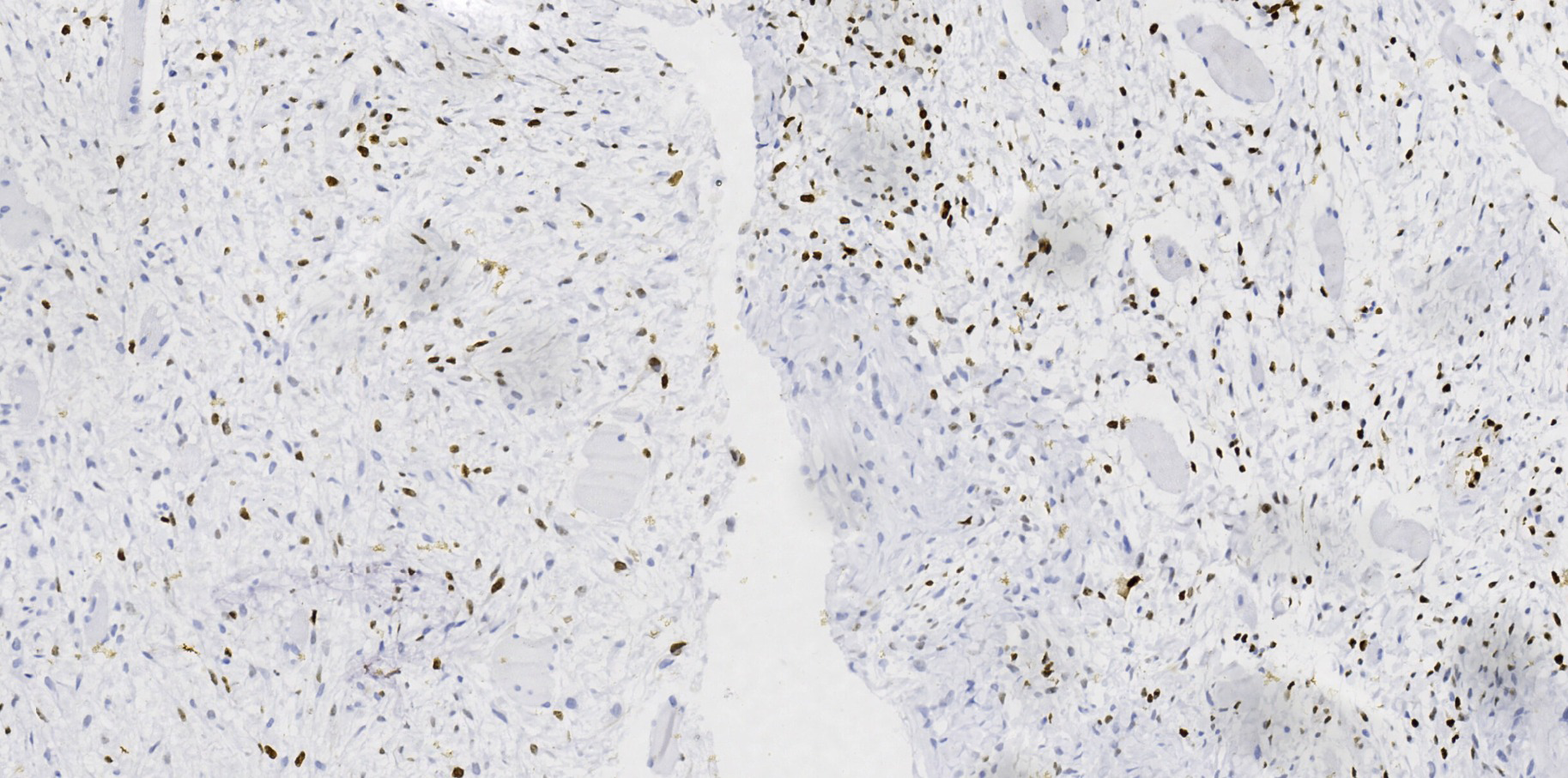

Supplement: Supplementary file 1 [file jpm-12-02079-s001.zip › Supplemental Materials S3/S3-Ki67.tif]

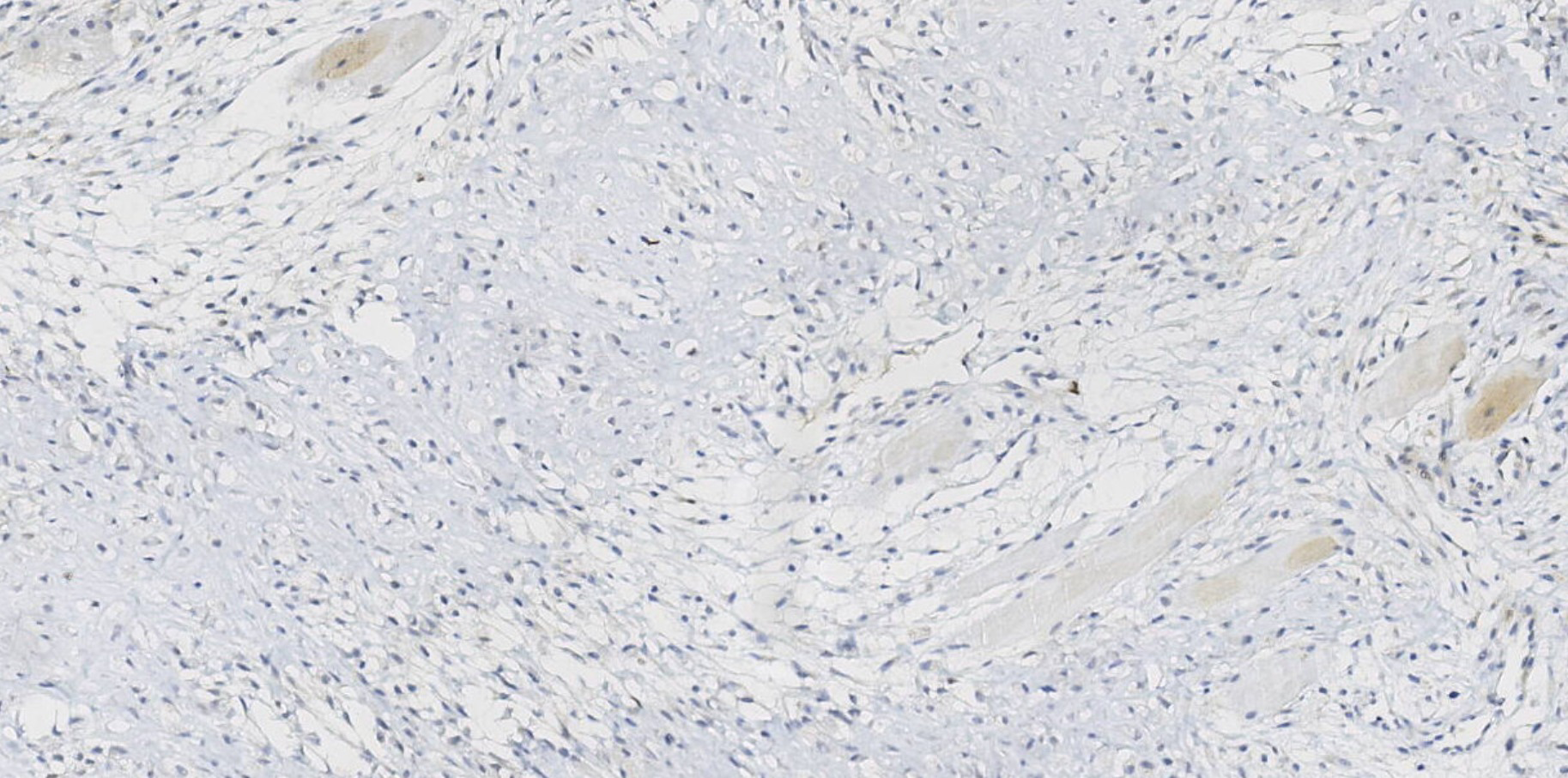

Supplement: Supplementary file 1 [file jpm-12-02079-s001.zip › Supplemental Materials S3/S3-MDM2.tif]

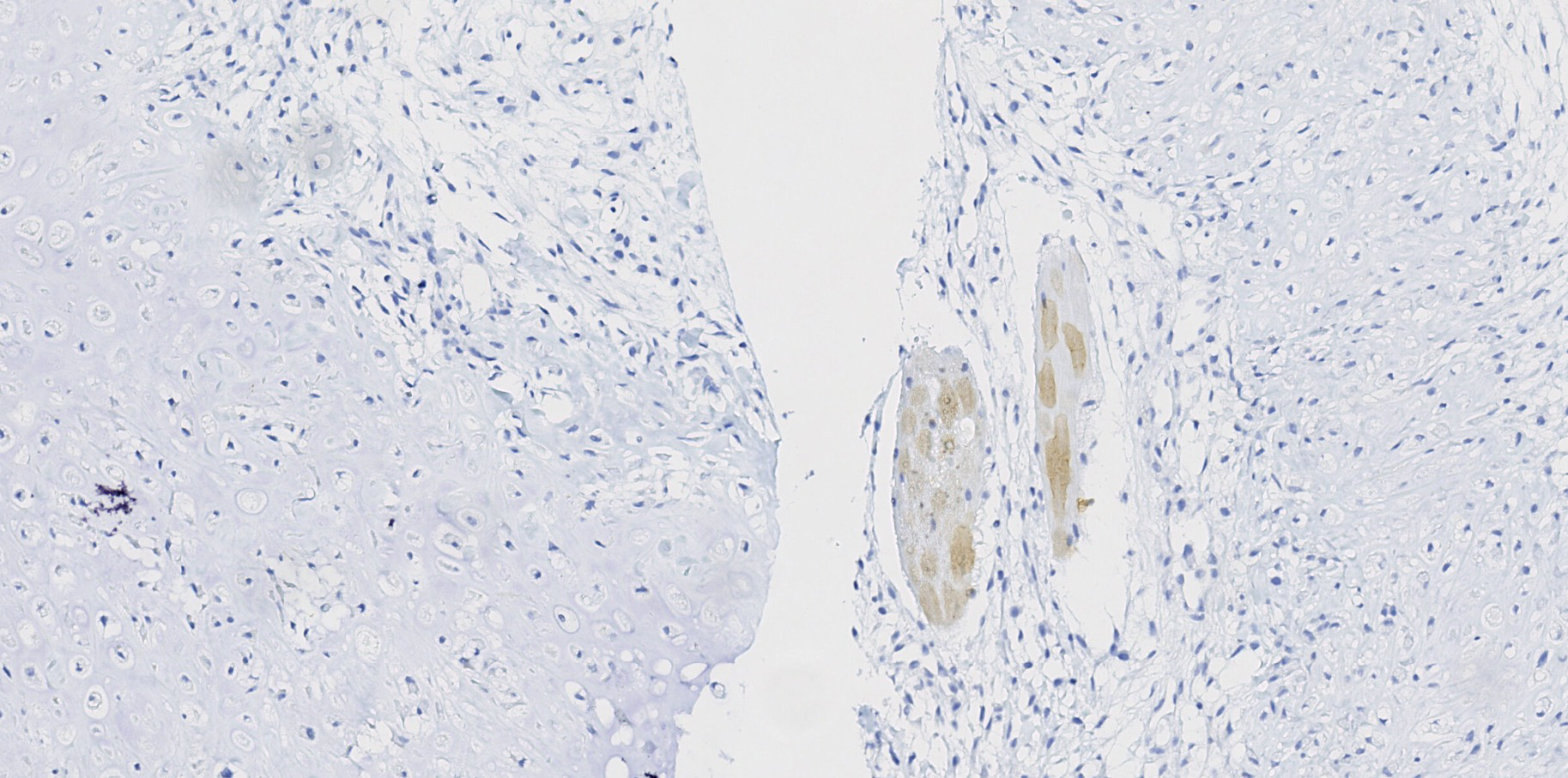

Supplement: Supplementary file 1 [file jpm-12-02079-s001.zip › Supplemental Materials S3/S3-Melan-A.tif]

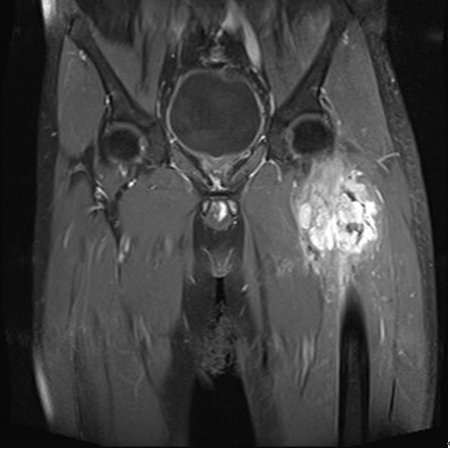

Supplement: Supplementary file 1 [file jpm-12-02079-s001.zip › Supplemental Materials S3/S3-MRI 1.tif]

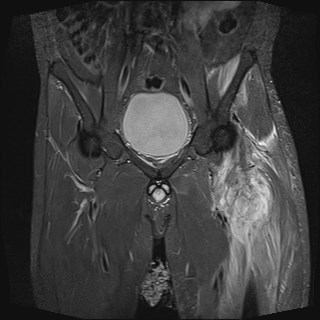

Supplement: Supplementary file 1 [file jpm-12-02079-s001.zip › Supplemental Materials S3/S3-MRI 2.tif]

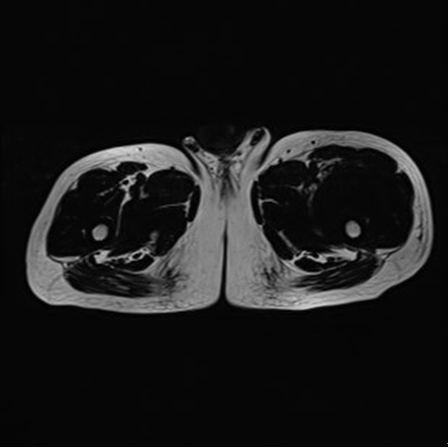

Supplement: Supplementary file 1 [file jpm-12-02079-s001.zip › Supplemental Materials S3/S3-MRI 3.tif]

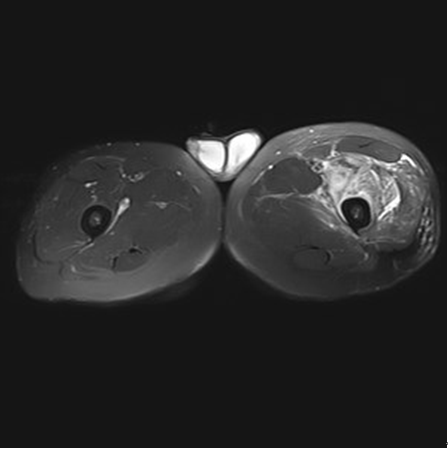

Supplement: Supplementary file 1 [file jpm-12-02079-s001.zip › Supplemental Materials S3/S3-MRI 4.tif]

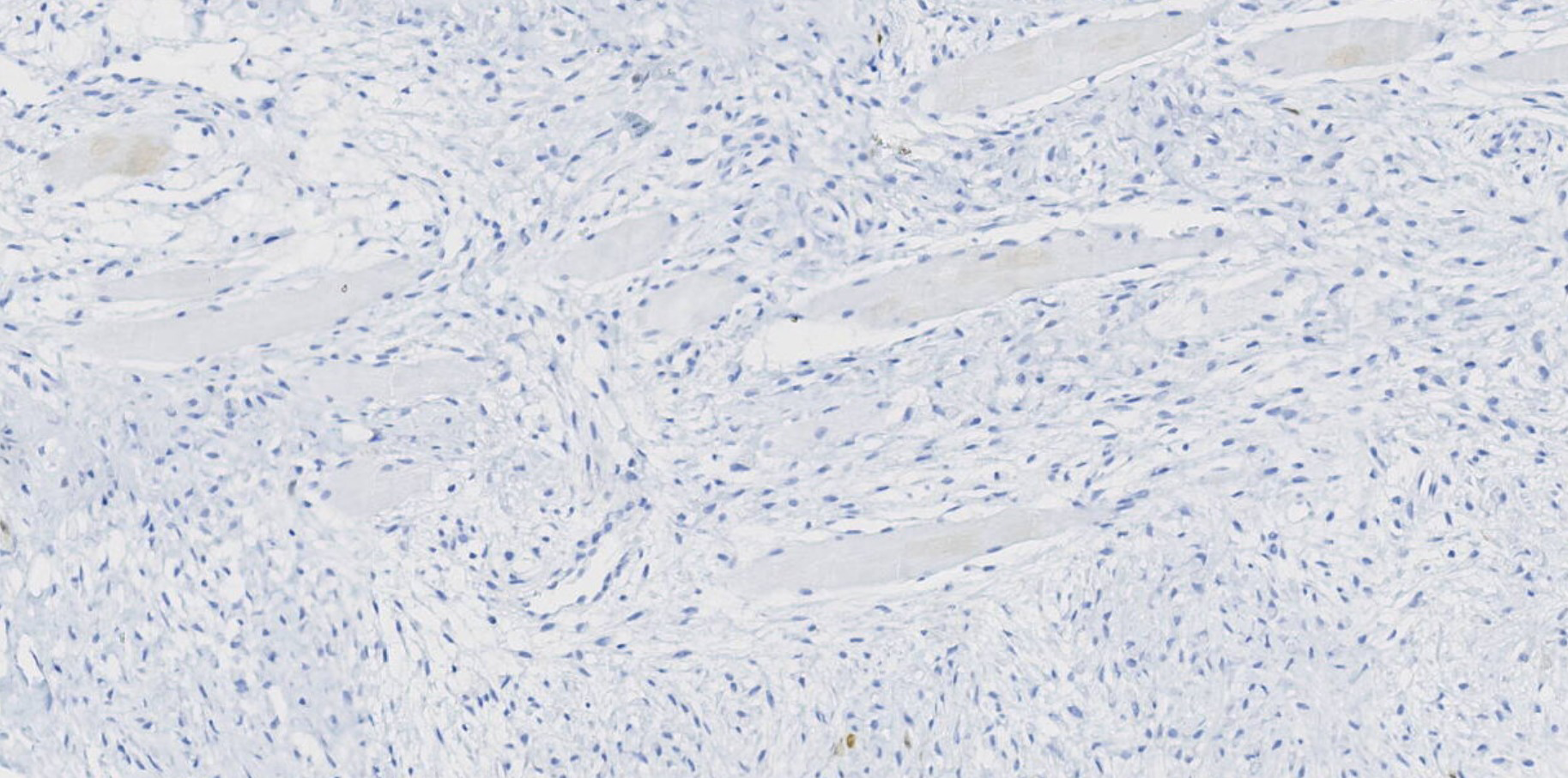

Supplement: Supplementary file 1 [file jpm-12-02079-s001.zip › Supplemental Materials S3/S3-P16.tif]

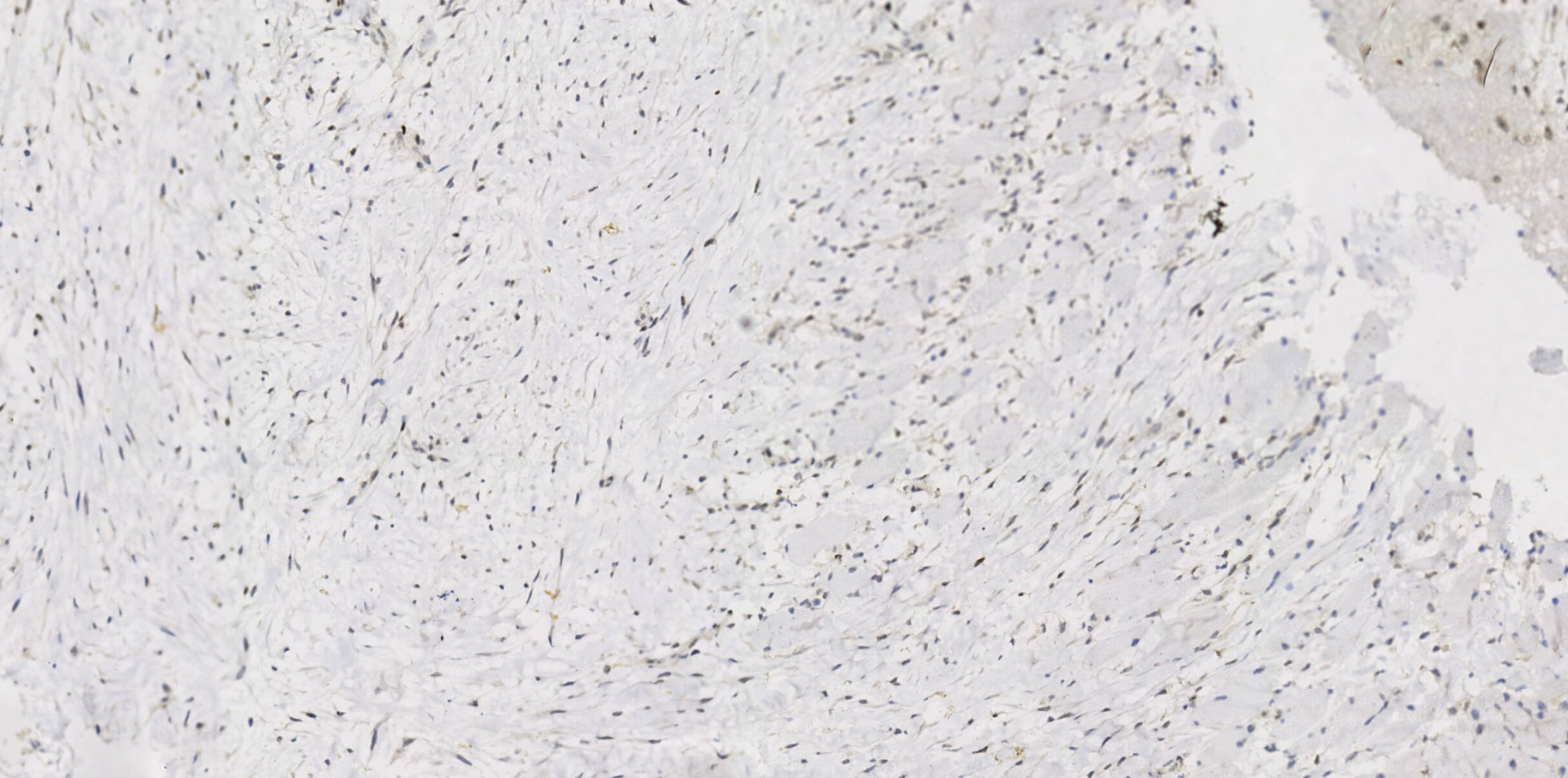

Supplement: Supplementary file 1 [file jpm-12-02079-s001.zip › Supplemental Materials S3/S3-P53.tif]

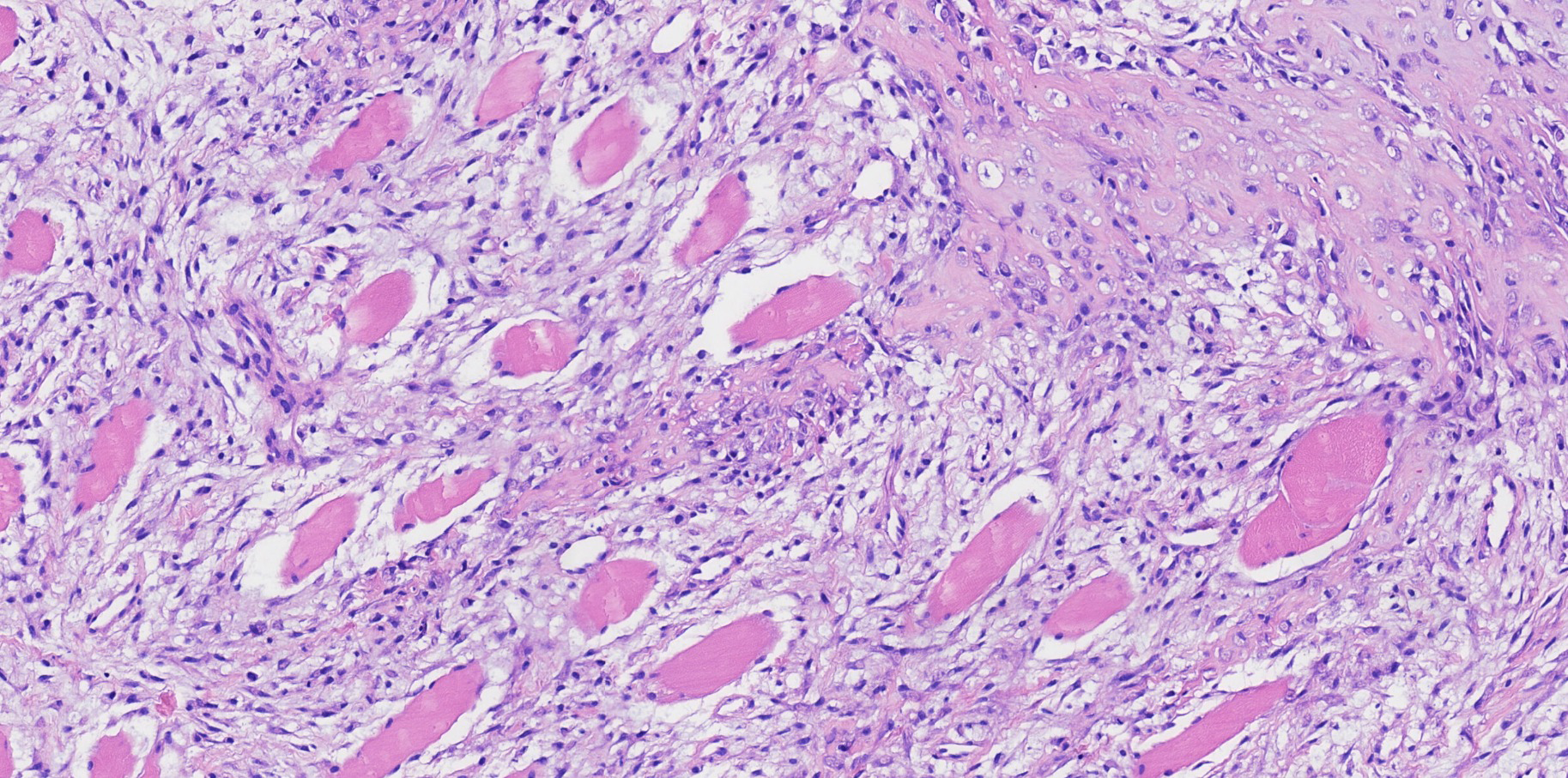

Supplement: Supplementary file 1 [file jpm-12-02079-s001.zip › Supplemental Materials S3/S3-Paraffin section HE100+.tif]

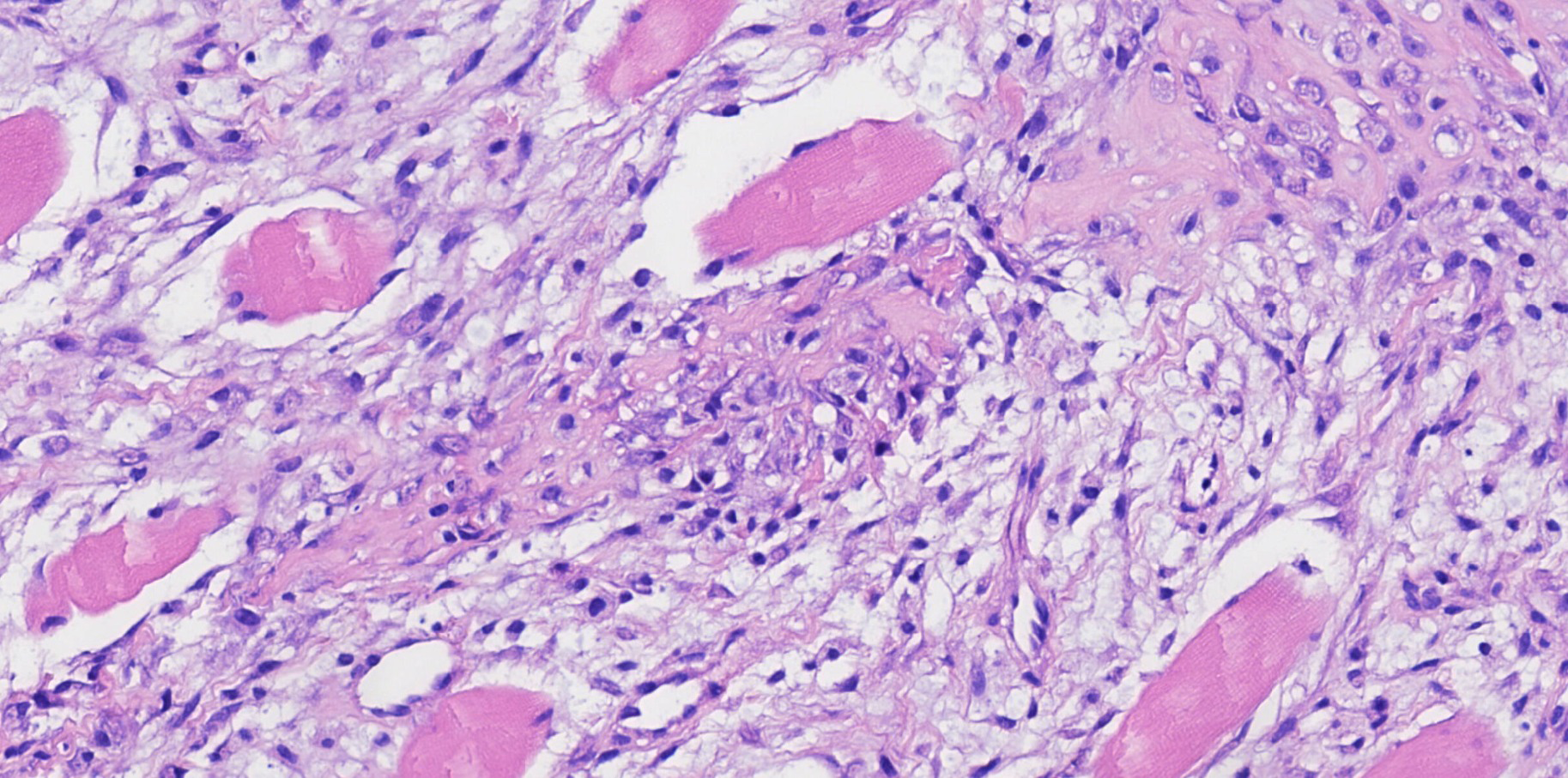

Supplement: Supplementary file 1 [file jpm-12-02079-s001.zip › Supplemental Materials S3/S3-Paraffin section HE200+.tif]

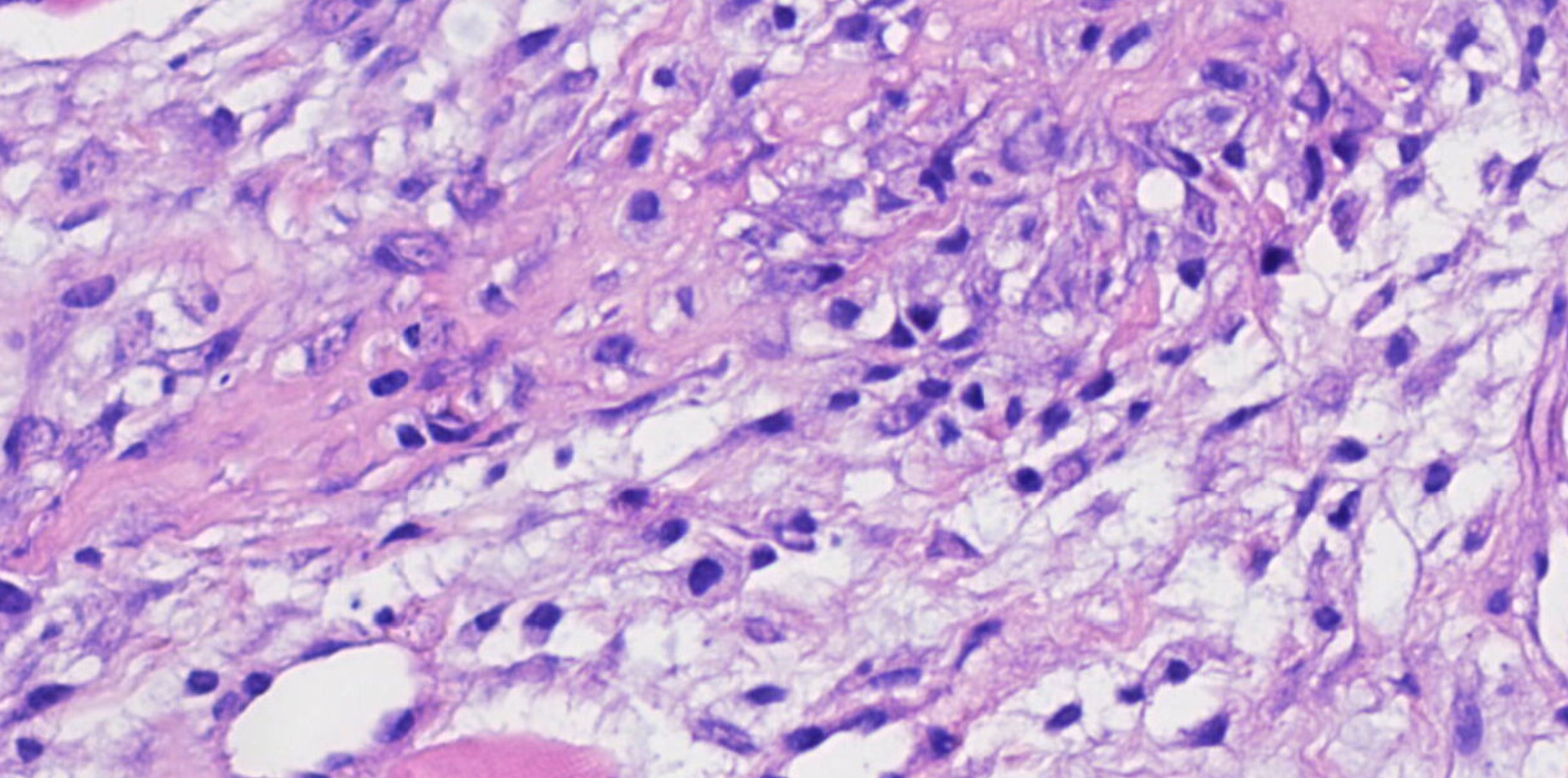

Supplement: Supplementary file 1 [file jpm-12-02079-s001.zip › Supplemental Materials S3/S3-Paraffin section HE400+.tif]

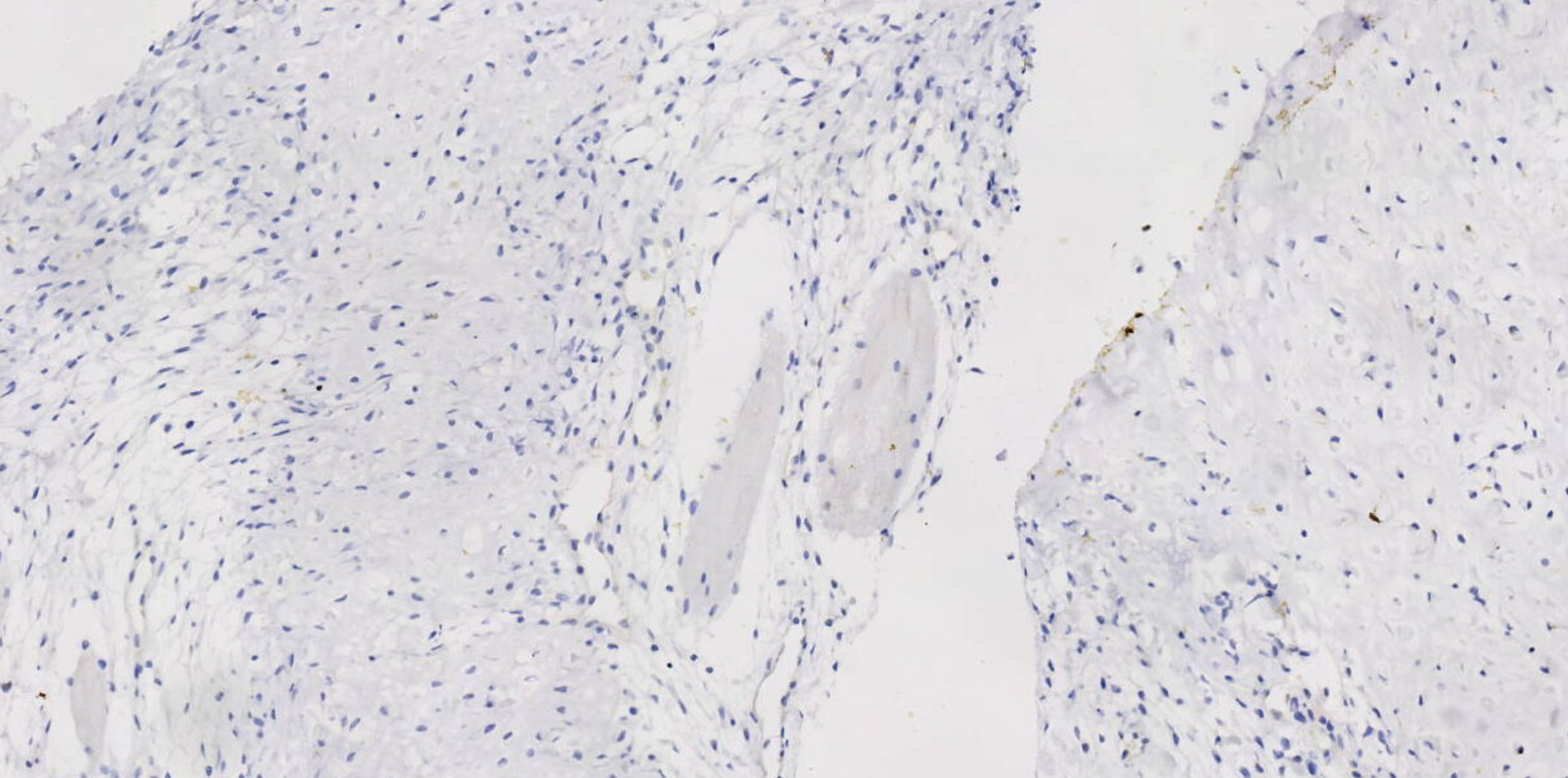

Supplement: Supplementary file 1 [file jpm-12-02079-s001.zip › Supplemental Materials S3/S3-S100.tif]

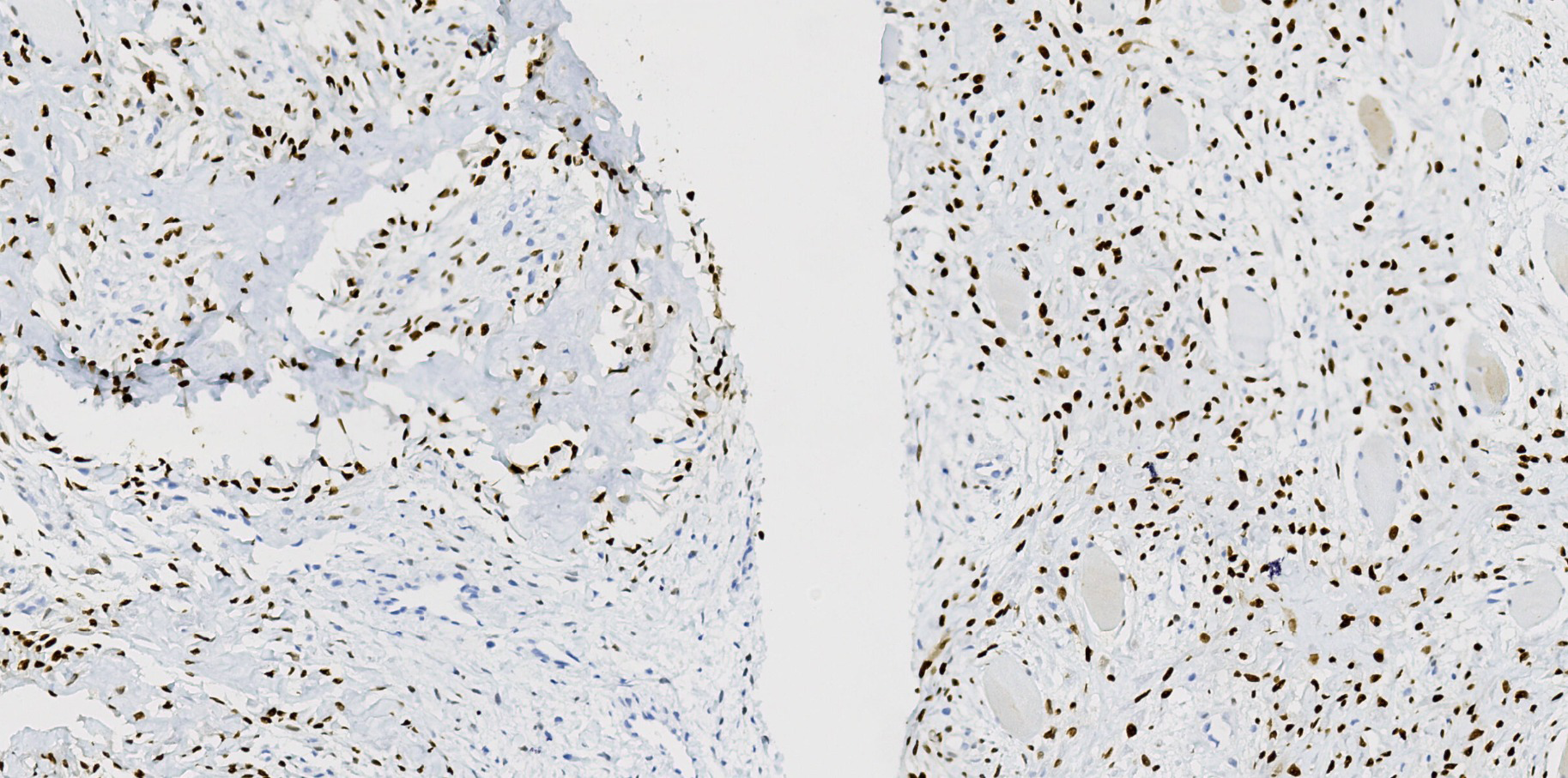

Supplement: Supplementary file 1 [file jpm-12-02079-s001.zip › Supplemental Materials S3/S3-SATB2.tif]

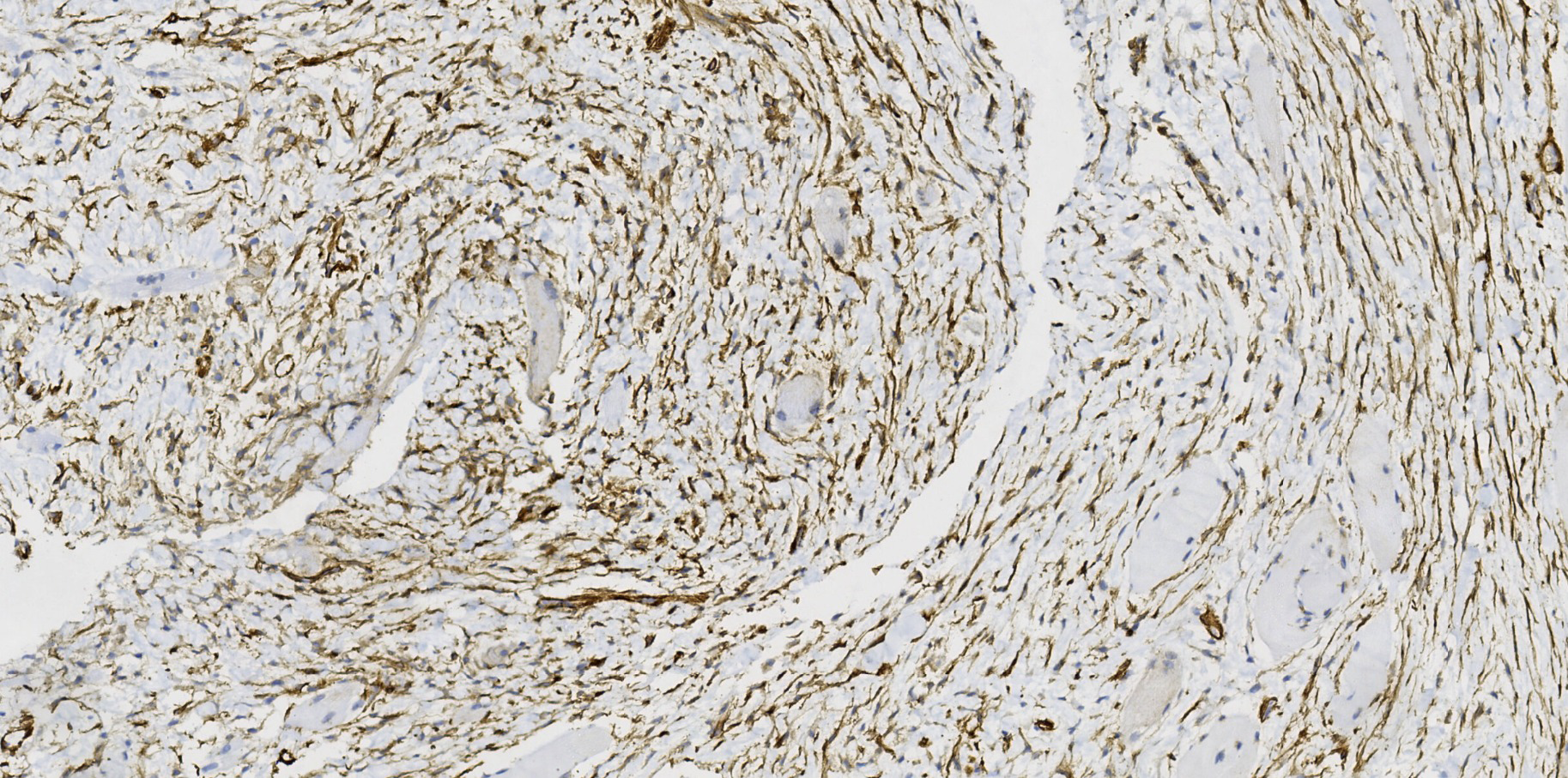

Supplement: Supplementary file 1 [file jpm-12-02079-s001.zip › Supplemental Materials S3/S3-SMA.tif]

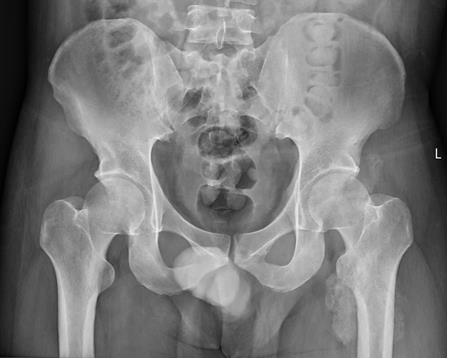

Supplement: Supplementary file 1 [file jpm-12-02079-s001.zip › Supplemental Materials S3/S3-X-ray 1.tif]

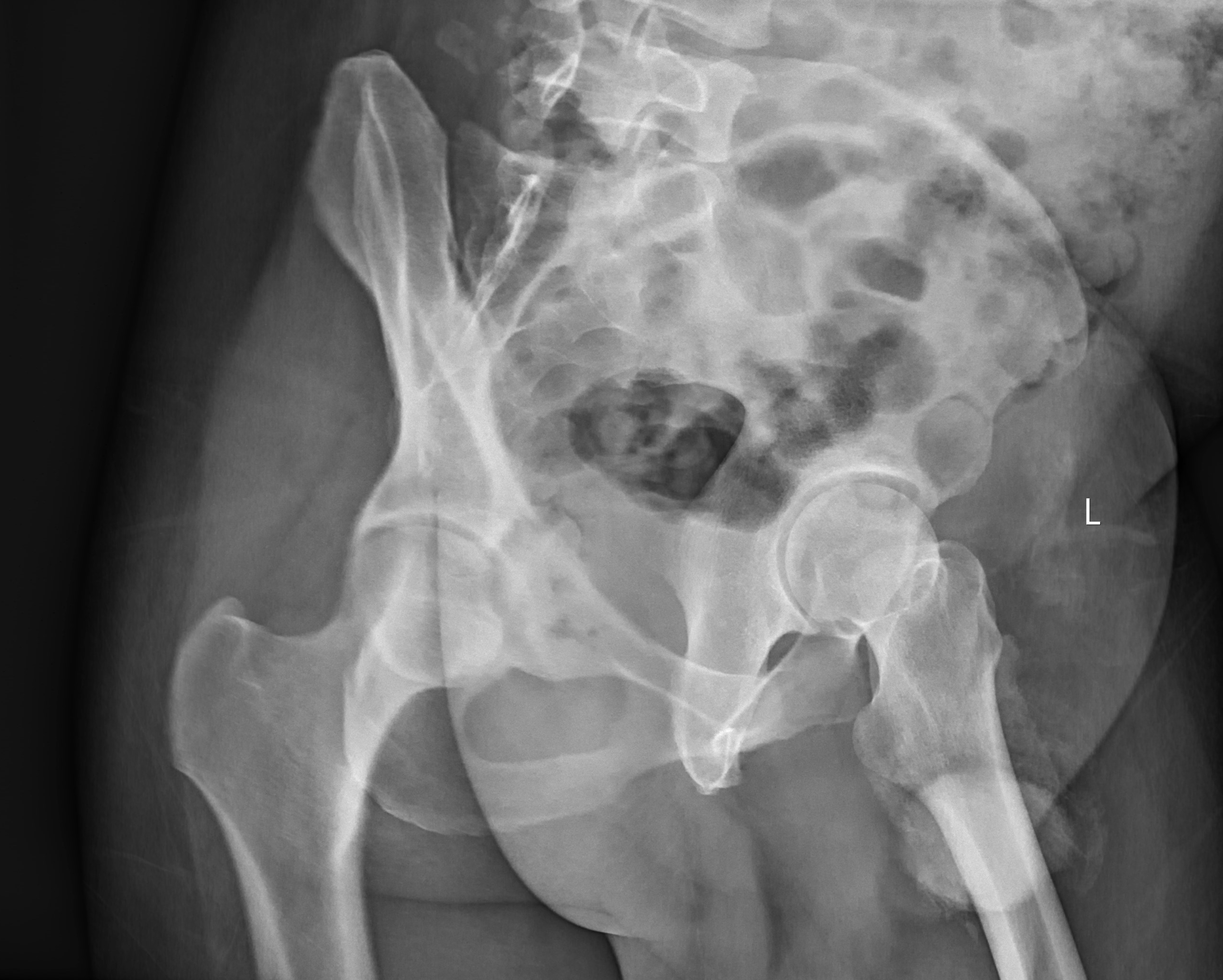

Supplement: Supplementary file 1 [file jpm-12-02079-s001.zip › Supplemental Materials S3/S3-X-ray 2.tif]
